# Description and Comparative Genomics of *Macrococcus caseolyticus* subsp. *hominis* subsp. nov., *Macrococcus goetzii* sp. nov., *Macrococcus epidermidis* sp. nov., and *Macrococcus bohemicus* sp. nov., Novel Macrococci From Human Clinical Material With Virulence Potential and Suspected Uptake of Foreign DNA by Natural Transformation

**DOI:** 10.3389/fmicb.2018.01178

**Published:** 2018-06-13

**Authors:** Ivana Mašlaňová, Zuzana Wertheimer, Ivo Sedláček, Pavel Švec, Adéla Indráková, Vojtěch Kovařovic, Peter Schumann, Cathrin Spröer, Stanislava Králová, Ondrej Šedo, Lucie Krištofová, Veronika Vrbovská, Tibor Füzik, Petr Petráš, Zbyněk Zdráhal, Vladislava Ružičková, Jiří Doškař, Roman Pantuček

**Affiliations:** ^1^Division of Genetics and Molecular Biology, Department of Experimental Biology, Faculty of Science, Masaryk University, Brno, Czechia; ^2^Czech Collection of Microorganisms, Department of Experimental Biology, Faculty of Science, Masaryk University, Brno, Czechia; ^3^Leibniz Institute Deutsche Sammlung von Mikroorganismen und Zellkulturen—German Collection of Microorganisms and Cell Cultures, Braunschweig, Germany; ^4^Central European Institute of Technology, Masaryk University, Brno, Czechia; ^5^Reference Laboratory for Staphylococci, National Institute of Public Health, Prague, Czechia; ^6^National Centre for Biomolecular Research, Faculty of Science, Masaryk University, Brno, Czechia

**Keywords:** *Macrococcus*, Gram-positive pathogens, prokaryotic transformation, methicillin resistance, Staphylococcal Cassette Chromosome (SCC), CRISPR-Cas, bacteriophage, plasmids

## Abstract

The genus *Macrococcus* is a close relative of the genus *Staphylococcus*. Whilst staphylococci are widespread as human pathogens, macrococci have not yet been reported from human clinical specimens. Here we investigated Gram-positive and catalase-positive cocci recovered from human clinical material and identified as *Macrococcus* sp. by a polyphasic taxonomic approach and by comparative genomics. Relevant phenotypic, genotypic and chemotaxonomic methods divided the analyzed strains into two separate clusters within the genus *Macrococcus*. Comparative genomics of four representative strains revealed enormous genome structural plasticity among the studied isolates. We hypothesize that high genomic variability is due to the presence of a *com* operon, which plays a key role in the natural transformation of bacilli and streptococci. The possible uptake of exogenous DNA by macrococci can contribute to a different mechanism of evolution from staphylococci, where phage-mediated horizontal gene transfer predominates. The described macrococcal genomes harbor novel plasmids, genomic islands and islets, as well as prophages. Capsule gene clusters, intracellular protease, and a fibronectin-binding protein enabling opportunistic pathogenesis were found in all four strains. Furthermore, the presence of a CRISPR-Cas system with 90 spacers in one of the sequenced genomes corresponds with the need to limit the burden of foreign DNA. The highly dynamic genomes could serve as a platform for the exchange of virulence and resistance factors, as was described for the methicillin resistance gene, which was found on the novel composite SCC*mec*-like element containing a unique *mec* gene complex that is considered to be one of the missing links in SCC evolution. The phenotypic, genotypic, chemotaxonomic and genomic results demonstrated that the analyzed strains represent one novel subspecies and three novel species of the genus *Macrococcus*, for which the names *Macrococcus caseolyticus* subsp. *hominis* subsp. nov. (type strain CCM 7927^T^ = DSM 103682^T^), *Macrococcus goetzii* sp. nov. (type strain CCM 4927^T^ = DSM 103683^T^), *Macrococcus epidermidis* sp. nov. (type strain CCM 7099^T^ = DSM 103681^T^), and *Macrococcus bohemicus* sp. nov. (type strain CCM 7100^T^ = DSM 103680^T^) are proposed. Moreover, a formal description of *Macrococcus caseolyticus* subsp. *caseolyticus* subsp. nov. and an emended description of the genus *Macrococcus* are provided.

## Introduction

The genus *Macrococcus*, the closest relative of staphylococci, is currently comprised of eight species (Parte, 2014) which are commonly isolated from animal skin (ponies, horses, cows, llamas, and dogs), from milk or meat products (Kloos et al., 1998; Mannerová et al., 2003; Gobeli Brawand et al., 2017). The phylogenetic relationship to the genus *Staphylococcus* was proven by a genomic study that shows that almost 65% of the predicted genes of *Macrococcus caseolyticus* have the highest homology with genes of staphylococci, and the two genera share many metabolic pathways (Baba et al., 2009). Phylogenetically and genotypically, macrococci are similar to oxidase-positive, novobiocin resistant staphylococci from the *Staphylococcus sciuri* complex (Götz et al., 2006; Švec et al., 2016; Christo-Foroux et al., 2017).

Although none of the species of the genus *Macrococcus* are thought to be human pathogens, there are reports of macrococci associated with animal infections (Gómez-Sanz et al., 2015; Hansen et al., 2015; Cotting et al., 2017). These findings suggest the possible pathogenicity of macrococci. Furthermore, the identification of methicillin-resistant strains of macrococci carrying the staphylococcal cassette chromosome (SCC) in their genome reveals a potential broad host-range dissemination of this resistance element between the genera *Macrococcus* and *Staphylococcus* (Baba et al., 2009; Rubin and Chirino-Trejo, 2010; Tsubakishita et al., 2010; Cicconi-Hogan et al., 2014; Gómez-Sanz et al., 2015; Micheel et al., 2015).

SCC carrying a *mec* complex with *mecA* or *mecC* genes has been described in staphylococci (Paterson et al., 2014; Pantuček et al., 2017). The methicillin resistance of macrococci has been associated with the presence of the *mecB* gene, a distantly related *mec*A homolog. Tsubakishita et al. (2010) identified the *mec* complex (*mecI–mecRI–mecB–blaZ*) to be part of transposon *Tn*6045 integrated downstream of the *orf* X gene on the chromosome of *M. caseolyticus* JCSC7096. The adjacent chromosome cassette recombinase (*ccr*) complex co-excises together with the *mec* complex to form an extrachromosomal SCC*mec* element. The potential for horizontal gene transfer (HGT) is also supported by the identification of the *mec* complex-carrying transposon *Tn*6045 on plasmid pMCCL2 (Baba et al., 2009). Similar elements were described in other *M. caseolyticus* strains (Tsubakishita et al., 2010) and an SCC*mec* element independent of *Tn*6045 was identified in a chromosome of the recently described *Macrococcus canis* (Gómez-Sanz et al., 2015; Gobeli Brawand et al., 2018).

A new allele of the *mec* gene (*mecD*) conferring resistance to all β-lactam antibiotics, including cephalosporins that are used to treat MRSA infections, was recently discovered in a methicillin-resistant veterinary strain *M. caseolyticus* IMD0819 (Schwendener et al., 2017). The *mecD* gene was localized on a genomic resistance island, whose structure resembles the *Staphylococcus aureus* resistance island. The genomic island contains an integrase gene that shares a close common ancestor with integrases of the genus *Bacillus*. This might indicate possible HGT between species of the families *Bacillaceae* and *Staphylococcaceae* (Schwendener et al., 2017).

Despite the evidence that macrococci take part in HGT and might serve as a reservoir of resistance and virulence determinants for veterinary pathogenic bacteria, very little is known about their genome plasticity and mobile genetic elements (MGE). So far no macrococci from human clinical specimens have been described. In this study, a polyphasic taxonomic approach using phenotypic and genotypic methods has been used to clarify the taxonomic position of seven *Macrococcus* sp. isolates originating from human clinical material. In four strains, the whole genome sequences were obtained and analyzed with a focus on various MGE and possible virulence factors. In addition, this work contributes to our understanding of the evolutionary relationships of bacterial pathogens in the family *Staphylococcaceae*.

## Materials and methods

### Bacterial strains

Seven Gram-stain positive, catalase-positive cocci isolated from human clinical material in different routine clinical laboratories (Table 1) were sent to the Reference Laboratory for Staphylococci (Prague, Czech Republic) for identification. Cells grown on TSA agar (Oxoid) were used for biotyping and genotyping analyses. All isolates were maintained on glass beads at −70°C in BHI broth supplemented with 15% glycerol (v/v). Type strains representing known *Macrococcus* species and reference *M. caseolyticus* strains CCM 8657, CCM 8658, P262, and P885 were obtained from the Czech Collection of Microorganisms (Masaryk University, Brno). *Macrococcus canis* DSM 101690^T^ (= KM45013^T^) was obtained from the Leibniz-Institut DSMZ-Deutsche Sammlung von Mikroorganismen und Zellkulturen GmbH (DSMZ) and deposited in the Czech Collection of Microorganisms under strain number CCM 8748^T^ in this work.

**Table 1:** Source of human *Macrococcus* spp. strains characterized in this study.

**Species/subspecies**	**Strain number**	**Year of isolation**	**Locality in the Czech Republic**	**Specimen**	**Patients' age range (years)**	**Diagnosis**
*M. caseolyticus* subsp. *hominis* subsp. nov.	CCM 7927^T^	2003	Příbram	Vaginal smear	40–45	Acute vaginitis
*M. caseolyticus* subsp. *hominis* subsp. nov.	CCM 7928	2003	Příbram	Wound smear	30–35	A wound from surgery after ankle fracture
*M. caseolyticus* subsp. *hominis* subsp. nov.	P862	2003	Příbram	Vaginal smear	40–45	Chronic vulvitis
*M. caseolyticus* subsp. *hominis* subsp. nov.	P865	2003	Příbram	Urine	66–70	Cervicitis
*M. goetzii* sp. nov.	CCM 4927^T^	2000	České Budějovice	Nail feet smear	30–35	Mycose
*M. epidermidis* sp. nov.	CCM 7099^T^	2001	České Budějovice	Skin smear	66–70	Mycose
*M. bohemicus* sp. nov.	CCM 7100^T^	2003	Strakonice	Knee wound	80–85	A traumatic wound

### Phenotypic characterization

Extensive phenotypic characterization using API 50CH, API ID 32 Staph and API ZYM (bioMérieux) commercial kits, phenotypic fingerprinting using the Biolog system with the GP2 MicroPlate identification test panel (Biolog) and conventional biochemical, physiological and growth tests relevant for the genus *Staphylococcus* were done as described previously (Mannerová et al., 2003; Pantuček et al., 2005, 2013). Haemolysis was examined on Columbia blood agar (Oxoid) supplemented with 7% sheep blood at 37°C (pH 7.2) after 24 h of incubation in a synergy test with the β-haemolysin-producing strain *Staphylococcus pseudintermedius* CCM 4710. The antibiotic resistance pattern was tested by the disc diffusion method on Mueller-Hinton agar (Oxoid). Antimicrobial susceptibility disks generally used for Gram-positive cocci were applied: ampicillin (10 μg), cefoxitin (30 μg), ceftazidime (10 μg), cephalothin (30 μg), ciprofloxacin (5 μg), clindamycin (2 μg), erythromycin (15 μg), gentamicin (10 μg), chloramphenicol (30 μg), imipenem (10 μg), kanamycin (30 μg), neomycin (10 μg), novobiocin (5 μg), oxacillin (1 μg), penicillin G (1 IU), cotrimoxazole (25 μg), tetracycline (30 μg), vancomycin (30 μg). EUCAST/CLSI standards were strictly followed for cultivation and inhibition zone diameter readings (CLSI, 2015; EUCAST, 2017).

### Transmission electron microscopy

The surface of plates containing bacterial cultures were washed off and resuspended in distilled water. A 200-mesh formvar-coated grid was placed on the drop of suspension for 20 min. Bacterial cells located on the grid were negatively-stained with 2% ammonium molybdate and treated with UV light. A T Morgagni 268D Philips (FEI Company) transmission electron microscope was used to visualize bacterial cells.

Phage lysate obtained after UV-light induction was centrifuged at 4,500 × g for 30 min at 4°C and filtered through 0.45 μm pore-sized polyethersulfone syringe filters (Techno Plastic Products) to remove bacterial debris. Phages were pelleted by centrifugation at 54,000 × g for 2.5 h at 4°C in a JA-30.50 Ti rotor (Beckman). Phage pellet resuspended in phage buffer (50 mM Tris.Cl pH 8.0, 10 mM CaCl_2_, 10 mM NaCl) was overlaid onto a preformed CsCl (Sigma) density gradient (1 mL of each 1.45 g/mL 1.50 g/mL, 1.70 g/mL of CsCl in phage buffer) and centrifuged at 194,000 × g for 4 h at 12°C using a SW 55 Ti rotor (Beckman). The fraction containing phages was collected and dialyzed against an excess of phage buffer at 4°C. For electron microscopy the sample was 10x concentrated by centrifugation, fixed by 2.5% (w/v) glutaraldehyde and deposited for 2 min on carbon-coated 400 mesh copper grids. The grids were washed twice on a drop of deionized water for 10 s and stained with 2% (w/v) uranyl acetate. The samples were analyzed by Tecnai F20 (Thermo Scientific) transmission electron microscope operated at 120 kV.

### Phylogenetic analysis using 16s rRNA and housekeeping genes

The extraction of genomic DNA by phenol-chloroform from cells treated with mutanolysin (Sigma-Aldrich), lysostaphin (Ambi Products LLC), and achromopeptidase (Sigma-Aldrich) was done as described previously (Gevers et al., 2001). Initial 16S rRNA gene amplification by PCR and partial 16S rRNA gene sequencing was performed as described previously by Mannerová et al. (2003). The complete 16S rRNA gene sequence was subsequently extracted from whole-genome shotgun (WGS) data using RNAmmer version 1.2 (Lagesen et al., 2007) and compared with those of other taxa of the genus *Macrococcus*, available in the GenBank database. Pairwise sequence alignment and calculation of similarity values was carried out by a global alignment algorithm, implemented at the EzBioCloud database (Yoon et al., 2017). The evolutionary history was inferred by the neighbor-joining and maximum likelihood methods using bootstrap values based on 1,000 replications with the software MEGA version 7 (Kumar et al., 2016). In addition, phylogenetic analysis was carried out using concatenated partial gene sequences and the corresponding proteins of the *hsp60, rpoB, tuf* , *dnaJ, gap*, and *sod* genes extracted from whole-genome sequencing data of representative strains analyzed in this study and reference sequences of *M. canis* CCM 8748^T^ (Gobeli Brawand et al., 2018) and *S. sciuri* DSM 20345^T^ (GenBank accession no. LEOS01000000).

### MALDI-TOF MS analysis

The samples were prepared by the standard extraction protocol described in detail by Freiwald and Sauer (2009). Approximately 5–10 mg of each culture was treated with 1.2 ml of 75% ethanol. After centrifugation and removal of the supernatant, cells were extracted with 25 μl of 70% formic acid followed by the addition of 25 μl of acetonitrile and vortexing at 2,000 rpm for 1 min. The supernatant after centrifugation was deposited in three positions (wells) of the sample plate in volumes of 0.3 μl and, after drying at room temperature, overlaid with 0.3 μl of the MALDI matrix solution. For identification purposes, alpha-cyano-4-hydroxycinnamic acid (CHCA) in water:acetonitrile:TFA (47.5:50:2.5, v/v) mixture was used as a MALDI matrix. For obtaining signals used for cluster analysis, the matrix solution was composed of 12.5 mg/ml ferulic acid in water:acetonitrile:formic acid (50:33:17, v/v), as proposed by Madonna et al. (2000). MALDI-TOF mass spectra were obtained using an Ultraflextreme instrument (Bruker Daltonics) operated in linear positive mode under FlexControl version 3.4 software. Nine independent mass spectra were acquired per sample (three mass spectra per well). Signals present in at least seven out of the nine mass spectra were taken into account. Mass spectra were processed using FlexAnalysis (version 3.4; Bruker Daltonics) and BioTyper software (version 3.1; Bruker Daltonics) supplemented with a database of 6903 reference entries (version 7.0.0.0). The mutual similarity between individual mass spectra of the strains and the BioTyper database entries were expressed in the form of log(scores) obtained using the default settings of the BioTyper software. A MALDI-TOF mass spectra-based dendrogram was constructed using the Pearson's product moment coefficient as a measure of similarity and the unweighted pair group average linked method (UPGMA) as a grouping method.

### Whole cell protein fingerprinting

A comparison of whole-cell protein profiles was carried out using an Agilent 2100 Bioanalyzer system with a Protein 230 kit (Agilent Technologies) and numerical analysis of the obtained fingerprints with the software BioNumerics version 7.6 (Applied Maths) was performed as described previously (Švec et al., 2015).

### Genotypic analysis by fingerprinting techniques

Rep-PCR fingerprinting with the (GTG)_5_ primer was performed as described previously (Švec et al., 2010). The automatic ribotyping with the *Eco*RI restriction endonuclease was performed using the RiboPrinter Microbial Characterization System (DuPont Qualicon) in accordance with the manufacturer's instructions. Numerical analysis of obtained fingerprints and dendrograms construction was done using the software BioNumerics version 7.6. The ribotype patterns were imported into BioNumerics using the load samples import script provided by the manufacturer. Pulsed-field gel electrophoresis (PFGE) using *Sma*I macrorestriction pattern analysis was performed as described by Pantuček et al. (1996).

### Chemotaxonomic analyses

The fatty acids methyl esters analysis (FAME) was performed with cells growing on BBL Tryptone Soya Agar (TSA) plates (Becton, Dickinson and Company) at 37°C for 24 hrs. The extraction of fatty acid methyl esters was performed according to the standard protocol (Sasser, 1990). Cellular fatty acid extracts were analyzed with an Agilent 7890B gas chromatograph by using the Sherlock Identification System (MIS, version 6.2B, MIDI database: RTSBA 6.21, MIDI Inc.).

Biomass and freeze-dried cells for chemotaxonomic studies were obtained after growing on TSA (Oxoid) at 36.5°C for 24 h. Peptidoglycan was isolated and its structure was analyzed according to published protocols (Schumann, 2011). Menaquinones were extracted from lyophilized cells according to the method of Collins et al. (1977) and analyzed by HPLC (Shimadzu LC 20A) as reported earlier (Groth et al., 1996).

### Genome sequencing and bioinformatics analysis

The concentration of extracted DNA was estimated with a Qubit 2.0 Fluorometer using a Qubit dsDNA BR assay kit (Invitrogen). WGS sequencing was performed using an Ion Torrent™ Personal Genome Machine (Thermo Fisher Scientific). The purified genomic DNA was used for preparing a 400-bp sequencing library with an Ion Plus Fragment Library Kit (Thermo Fisher Scientific). The sample was loaded onto a 316v2 chip and sequenced using an Ion PGM Hi-Q View sequencing kit (Thermo Fisher Scientific). Quality trimming of the reads was performed with the Ion Torrent Suite Software version 5.0.4 with default settings. The assembly computation and error correction was performed using the Assembler SPAdes version 3.11.1 pipeline (Nurk et al., 2013) with recommended parameters for Ion Torrent data. Contigs were then re-ordered according to the reference genome *M. canis* CCM 8748^T^ (Gobeli Brawand et al., 2018) using MauveContigMover (Rissman et al., 2009). Ordering was evaluated using assembly graph visualized in Bandage (Wick et al., 2015). The plasmid contigs were extracted from WGS data based on higher coverage and content of typical plasmid-borne genes. The plasmid DNA was extracted using the NucleoSpin Plasmid Kit (Macherey-Nagel) according to the manufacturer's protocol with the modification that bacteria were lysed with lysostaphin (30 μg/ml, Ambi Products LLC) and with 4U of mutanolysin (Sigma-Aldrich) at 37°C. The complete plasmid sequencing was achieved by a primer-walking approach. Sequences were manipulated and inspected in the cross-platform bioinformatics software Ugene version 1.28.0 (Okonechnikov et al., 2012). For primal analysis, the genome was annotated using RAST (Aziz et al., 2008). Gene content was further examined by Protein BLAST (https://blast.ncbi.nlm.nih.gov), ISfinder (Siguier et al., 2006), IslandViewer 4 (Dhillon et al., 2015), and InterProScan (Jones et al., 2014). Protein sequences predicted by RAST annotation were clustered using web-based OrthoVenn with a default cutoff e-value of 1e^−5^ and inflation value of 1.5 (Wang et al., 2015), and were searched against the virulence factors database VFDB using an e-value of 1e^−4^ (Chen et al., 2016). The presence of CRISPR-Cas systems were tested using CRISPRone (Zhang and Ye, 2017).

### DNA homology studies

Cells were disrupted by using a Constant Systems TS 0.75 KW (IUL Instruments) and the DNA in the crude lysate was purified by chromatography on hydroxyapatite as described by Cashion et al. (1977). DNA-DNA hybridization (DDH) was carried out as described by De Ley et al. (1970) taking into account the modifications described by Huss et al. (1983) using a model Cary 100 Bio UV/VIS-spectrophotometer equipped with a Peltier-thermostatted 6 × 6 multicell changer and a temperature controller with *in-situ* temperature probe (Varian). To evaluate the genomic similarity among type strains, an average nucleotide identity (ANI) and a digital DDH (dDDH) were determined. The dDDH values were calculated using web-based genome-to-genome distance calculator (GGDC) version 2.1 (Meier-Kolthoff et al., 2013). Cluster analysis based on ANI values and a heat-map of ANI identity were obtained using the Python module PYANI script (Pritchard et al., 2016).

### Bacteriophage analyses

The prophages were identified from WGS data by PHASTER (Arndt et al., 2016). Phage induction by UV-irradiation and/or mitomycin C, vertical one-dimensional SDS-PAGE and LC-MS/MS identification of capsid proteins were performed according to Zeman et al. (2017).

### Genbank accession numbers

The GenBank accession numbers for the 16S rRNA genes of isolates CCM 7927^T^, CCM 7928, P862, P865, CCM 4927^T^, CCM 7099^T^, and CCM 7100^T^ are MH044686-MH044692. The data from Whole Genome Shotgun projects of strains CCM 3540^T^, CCM 7927^T^, CCM 4927^T^, CCM 7099^T^, and CCM 7100^T^ have been deposited at DDBJ/ENA/GenBank under the accession numbers: PZJF00000000, MJBI00000000, MJBJ00000000, PZJH00000000, and PZJG00000000, respectively, and annotated by the NCBI Prokaryotic Genome Annotation Pipeline.

## Results and discussion

### Phenotypic characterization

All seven isolates originating from human clinical specimens (Table 1) were observed to be spherical or slightly irregular cocci, occurring predominantly in pairs and clusters, non-spore forming and non-motile. The measured values of their cell size ranged from 1.02 to 1.14 μm in diameter based on transmission electron microscopy (TEM) (Figure S1), i.e., cells that are larger than those of staphylococci. Microscopic and macroscopic morphology and the results of key biochemical and physiological tests relevant for Gram-positive cocci, namely positive catalase and oxidase activity and growth in the presence of 7% NaCl, resistance to bacitracin and novobiocin, but susceptibility to furazolidone and negative growth in the presence of 10% NaCl presumptively identified all seven isolates as *Macrococcus* sp.

### Phylogenetic analyses

**Figure 1:**
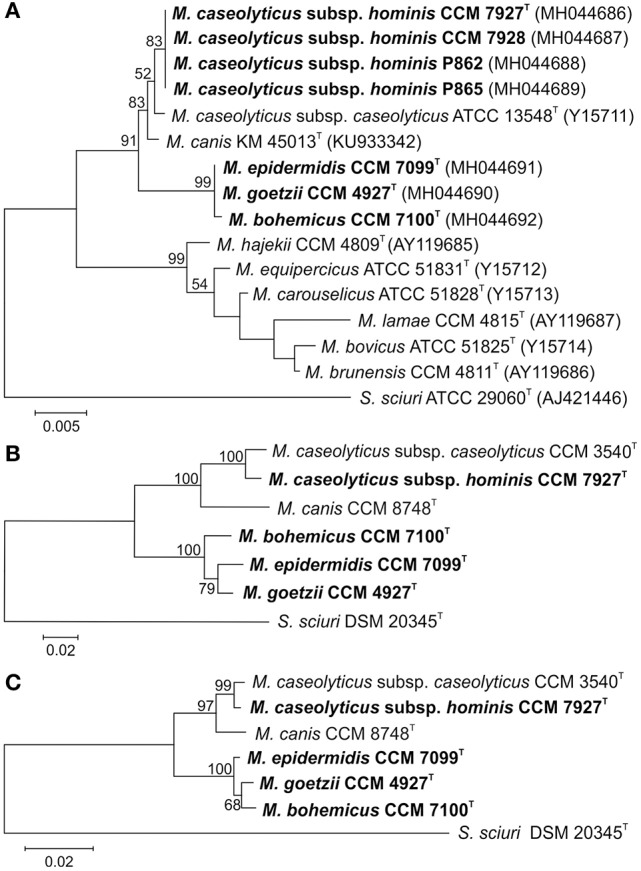
Phylogenetic trees showing the position of human macrococci within the genus *Macrococcus* based on **(A)** 16S rRNA gene sequence; **(B)** concatenated housekeeping genes *hsp60, rpoB, dnaJ, tuf* , *gap*, and *sodA*; and **(C)** concatenated housekeeping gene protein products. The sections of the gene sequences and their protein products correspond to the following gene coordinates of *Staphylococcus aureus* subsp. *aureus* NCTC 8325 (GenBank accession number NC_007795): 14 to 1608 for *hsp60*, 82 to 3552 for *rpoB*, 1 to 1116 for *dnaJ*, 7 to 1200 for *tuf* , 15 to 1026 for *gap*, and 2 to 599 for the *sod*A gene. The evolutionary history was inferred using the Maximum Likelihood method based on the Tamura-Nei model. The tree with the highest log likelihood is shown. Bootstrap probability values (percentages of 1,000 tree replications) greater than 50% are indicated at branch points. Bar indicates number of substitutions per nucleotide position. *Staphylococcus sciuri* type strain was used as an outgroup.

The obtained 16S rRNA gene sequences (Figure 1A) and multilocus sequence analyses (MLSA) based on 6 concatenated housekeeping genes, *hsp60, rpoB, tuf, gap, sod*A, and *dna*J (Figure 1B), and their protein products (Figure 1C) confirmed the phylogenetic position of the isolates within the genus *Macrococcus* and divided the strains into two separate groups. The phylogenetically closest known related species of both groups were strains from a phylogenetic clade including *M. caseolyticus* and *M. canis* (Figure 1). Strains CCM 7927^T^, CCM 7928, P862, and P865 representing group 1 had 100% reciprocal identity and 99.8% identity with the 16S rRNA sequence of *M. caseolyticus* CCM 3540^T^ and *M. canis* CCM 8748^T^. Group 2 formed a separate branch in the MLSA and included isolates CCM 4927^T^ and CCM 7099^T^, which have identical 16S rRNA gene sequences and are closely related to strain CCM 7100^T^, with 99.9% 16S rRNA gene sequence similarity. The next closest relatives of the group 2 strains were strain CCM 7927^T^ (99.1%), *M. caseolyticus* CCM 3540^T^ (99.0%) and *M. canis* CCM 8748^T^ (98.9%). Despite the high 16S rRNA gene sequence similarities, the MLSA data unambiguously confirmed the differentiation of the new isolates from the phylogenetically closely related known species *M. caseolyticus* and *M. canis* and differentiated all strains of group 2 from each other (Figures 1B,C).

### MALDI-TOF MS identification and whole cell protein profiling

The analyzed strains and reference type strains of all validly named *Macrococcus* spp. were subjected to a standard MALDI-TOF MS identification procedure including protein extraction, sample deposition with CHCA as the MALDI matrix, and comparison with a commercially available database (BioTyper, version 7.0.0.0; currently, from *Macrococcus* spp., only entries of *M. caseolyticus* exist in the database). Only strain CCM 3540^T^ yielded a log(score) greater than 2.000, indicating *M. caseolyticus* species identification with a high confidence. All group 1 strains produced scores between 1.700 and 1.999, which correspond to *M. caseolyticus* species identification with low confidence. The remaining strains did not yield mass spectra with significant similarity to any of the BioTyper database entries [log(scores) lower than 1.699]. The outputs of the cluster analysis based on signals obtained by MALDI-TOF MS analysis using ferulic acid as the MALDI matrix correlate with the other cluster analyses performed (Figure S2); group 1 strains formed a tight cluster with the *M. caseolyticus* CCM 3540^T^ strain and *M. canis* CCM 8748^T^ as the next closest relative, while group 2 strains formed another well-resolved group clearly distinguished from the reference type strains of *Macrococcus* spp.

Whole-cell protein fingerprinting also confirmed the separate position of the closely related group 1 strains and showed their relationship to *M. caseolyticus* strains. The three group 2 strains CCM 4927^T^, CCM 7099^T^, and CCM 7100^T^ were placed in a separate cluster, but these three strains exhibited more heterogeneous profiles. The closest profile to the group 2 strains was that of *M. canis* CCM 8748^T^. All the analyzed strains were clearly differentiated from all remaining representatives of known *Macrococcus* spp. (Figure S3).

### DNA fingerprinting analyses

The genomic relatedness among isolated strains and their clonal origin was studied by three DNA fingerprinting techniques, namely automated ribotyping (Figure S4), rep-PCR fingerprinting (Figure S5), and *Sma*I macrorestriction analysis determined by PFGE (Figure S6). Numerical analysis of the obtained fingerprints clearly separated group 1 strains from the remaining macrococci and showed genetic homogeneity within these four strains, revealing visually identical fingerprints by each of these techniques. The group 2 strains CCM 4927^T^, CCM 7099^T^, and CCM 7100^T^ were clearly differentiated from the remaining macrococci included in the analysis as well as from each other. The macrorestriction analysis of the strains showed diversity between group 1 and group 2 in their PFGE banding patterns. Group 1 strains exhibited fragments of similar lengths (from 5 kb to 190 kb) to *M. caseolyticus*, in contrast with the macrorestriction patterns of group 2 isolates, for which no significant similarity was found and which were ~20 kb to > 600 kb in size, resembling staphylococci (Figure S6).

### Chemotaxonomic analyses

All chemotaxonomic analyses of the isolates showed characteristics corresponding to those of the other species of the genus *Macrococcus*. FAME analysis (Table S1) showed that group 1 strains have a unique fatty acid composition compared *to M. caseolyticus* CCM 3540^T^ and *M. canis* CCM 8748^T^. The major fatty acids displayed by group 1 were identified as C_14:0_ (27.1%), C_16:0_ N alcohol (13.6%), C_16:1_ ω*11c* (11.5%), C_16:0_ (9.7%) and C_18:1_ ω*9c* (22.9%). The group 1 strains contained lower amounts of C_14:0_ iso (1.1%) and C_16:1_ ω*11c* (11.5%) compared to *M. caseolyticus* CCM 3540^T^ (7.9% and 29.5%, respectively); moreover, members of group 1 differed by a higher amount of C_14:0_ (27.1%), C _16:0_ (9.7%) and C_18:3_ ω*6c* (6,9,12) (6.7%) compared to *M. caseolyticus* CCM 3540^T^ (16.5%, 6.6% and 0%, respectively), confirming their divergence from this type strain.

Strains belonging to group 2 were found to have similar, but not identical fatty acid profiles. Common major fatty acids to all of them were C_15:0_ anteiso (12.3-17.5%), C_16:0_ (8.8-14.9%) and C_18:1_ ω*9c* (20.2-31.5%). Strain CCM 4927^T^ differs by the presence of two additional major fatty acids, namely C_14:0_ (11.8%) and C_16:1_ ω*9c* (11.8%). Strain CCM 7100^T^ was distinguishable by an additional major fatty acid C_16:0_ (14.9%). Strain CCM 7099^T^ differs by an additional major fatty acid C_18:0_ (12.7%) and by a low amount of C_14:0_ (3.6%) compared to the two other species in group 2. Members of group 2 were distinguishable from group 1, *M. caseolyticus* CCM 3540^T^ and *M. canis* CCM 8748^T^ based on the presence of C_15:0_ anteiso. Overall, the fatty acid profile of each member of group 2 exhibited a unique fatty acid pattern that represents an important distinctive feature.

The major menaquinone type obtained from the isolates was MK-6, which is in agreement with the data of Gobeli Brawand et al. (2017). The menaquinone profiles of representative strains were found to be as follows: CCM 7927^T^ [MK-6 and MK-7 (97:0.6)], CCM 4927^T^ [MK-6, MK-5, and MK-7 (98:1:0.1)], CCM 7099^T^ [MK-6 and MK-7 (96:0.5)], and CCM 7100^T^ [MK-6, MK-5, and MK-7 (97:2:0.3)].

The peptidoglycan type of the cell wall of strains CCM 7927^T^ and CCM 7099^T^ was determined to be A3α Lys-Gly_3_-Ser and so resembled the peptidoglycan type of majority *Macrococcus* spp. members (Schleifer, 2015), while the peptidoglycan type A3α L-Lys-Gly_1−2_ identified in strains CCM 4927^T^ and CCM 7100^T^ was different.

### Genome homology studies

Both wet-lab DNA-DNA hybridization (DDH) and whole-genome sequencing to compare genome-to-genome distances were performed in order to precisely clarify the taxonomic position of the analyzed human macrococcal isolates (Figure S7). After considering the recommendations of a threshold value of 70% DNA-DNA similarity for the definition of bacterial species by the *ad hoc* committee (Wayne et al., 1987) and 95–96% threshold ANI value (Richter and Rosselló-Móra, 2009; Meier-Kolthoff et al., 2013), the results showed that strain CCM 7927^T^ representing group 1 belongs to the species *M. caseolyticus*. In contrast, strains CCM 4927^T^, CCM 7099^T^, and CCM 7100^T^ were shown to be representatives of three distinct species, clearly differentiated from strains CCM 7927^T^ and *M. caseolyticus* CCM 3540^T^.

### Proposal of the new macrococcal taxa

Based on the above results of overall genome relatedness, a novel subspecies within *M. caseolyticus* and three novel species within the genus *Macrococcus* are proposed. Group 1 strains CCM 7927^T^, CCM 7928, P862, and P865, originating from various human clinical materials, formed a coherent cluster for which the name *M. caseolyticus* subsp. *hominis* subsp. nov. is proposed. In accordance with Rule 40d (formerly Rule 46) of the Prokaryotic Code (De Vos and Trüper, 2000; Parker et al., 2015) the remaining strains of *M. caseolyticus* represented by the type strain CCM 3540^T^ are reclassified as *M. caseolyticus* subsp. *caseolyticus* subsp. nov. Group 2 strains CCM 4927^T^, CCM 7099^T^ and CCM 7100^T^ represents three distinct species for which the names *Macrococcus goetzii* sp. nov. (type strain CCM 4927^T^) and *Macrococcus epidermidis* sp. nov. (type strain CCM 7099^T^), both inhabiting human skin, and *Macrococcus bohemicus* sp. nov. (type strain CCM 7100^T^), isolated from a knee wound, are proposed. The detailed protologues describing these four taxa are given below.

### Genome characteristics

Genomes of the strains CCM 7927^T^, CCM 4927^T^, CCM 7099^T^ and CCM 7100^T^ were shot-gun sequenced and compared with the reference genomes of *M. caseolyticus* subsp. *caseolyticus* subsp. nov. CCM 3540^T^, sequenced in this study, and *M. canis* CCM 8748^T^ (Gobeli Brawand et al., 2018). The sequencing and assembly statistics of the whole genome sequences obtained from the type strains are shown in Table 2. *Macrococcus caseolyticus* subsp. *hominis* subsp. nov. CCM 7927^T^ with a 2.1-Mb chromosome and G+C content of 36.8 mol% is closely related to the type strain *M. caseolyticus* subsp. *caseolyticus* subsp. nov. CCM 3540^T^, differing mainly in the content of variable genetic elements. All strains from group 2 exhibited larger chromosomes than *M. caseolyticus*, ranging from 2.3 to 2.6 Mb, with a notably lower G+C content of 34.0 to 34.2 mol% (Table 2). Their G+C content values lie below the 38–45 mol% range defined by Kloos et al. (1998) for the genus *Macrococcus* using the thermal denaturation method. A comparison of the G+C content in the core genome of the analyzed strains confirmed that this difference is not caused by the presence of accessory genes. Thus *M. goetzii* sp. nov.*, M. epidermidis* sp. nov. and *M. bohemicus* sp. nov. have similar G+C content to the members of the genus *Staphylococcus* (Schleifer and Bell, 2009).

**Table 2:** Summary of whole genome sequence characteristics of analyzed *Macrococcus* spp.

**Genome**	***M*. *caseolyticus* subsp. *caseolyticus* subsp. nov. CCM 3540^T^**	***M. caseolyticus* subsp. *hominis* subsp. nov. CCM 7927^T^**	***M. goetzii* sp. nov. CCM 4927^T^**	***M. epidermidis* sp. nov. CCM 7099^T^**	***M. bohemicus* sp. nov. CCM 7100^T^**
WGS Project no.	PZJF00000000	MJBI00000000	MJBJ00000000	PZJH00000000	PZJG00000000
Size (Mb)	2.06	2.10	2.59	2.48	2.30
No. of contigs (>200 bp)	42	37	33	31	29
N50	72 809	189 524	459 952	214 089	260 501
Mean Coverage	67	66	313	264	272
G+C content (mol%)	36.56	36.79	33.95	33.97	34.23
G+C content in core genome (mol%)	36.77	37.02	34.04	34.08	34.32
Coding sequences	2140	2149	2679	2588	2462
No. of prophages	1	1 (ϕMC1)	2 (ϕMG1, ϕMG2)	1 (ϕME1)	1 (ϕMB1)
SCC-like elements	ψSCC_CCM3540_	ψSCC_CCM7927_	MgCI-ψSCC*mec*_CCM4927_	MeCI-ψSCC_CCM7099_	MbCI-ψSCC_CCM7100_
other chromosomal/resistance island	1	1 (McRI_CCM7927_)	–	1 (MeCI_CCM7099_)	2 (MbRI-1_CCM7100_, MbRI-2_CCM7100_)
PICI (Phage-inducible chromosomal island)	3	–	*2* (MgCI-1_CCM4927_, MgCI-2_CCM4927_)	–	1 (MbCI_CCM7100_)
No. of plasmids	N/A	1 (pZKMH1)	1 (pZKMG1)	1 (pZKME1)	2 (pZKMB1, pZKMB2)
CRISPR	–	–	–	–	1 (type II-C)
virulence factors	N/A	*fbpA*^*c*^; *veg*^*d*^; *hly-III*^*e*^; *cap*^*f*^	*sdr* loci^*a*^; PIA^*b*^; *fbpA*^*c*^; *veg*^*d*^; *hly-III^*e*^; cap*^*f*^; *adsA*^*g*^	*sdr* loci^*a*^;PIA^*b*^; fbpA^*c*^; *veg*^*d*^; *hly-III*^*e*^; *cap*^*f*^	PIA^*b*^; *fbpA*^*c*^; *veg*^*d*^; *hly-III*^*e*^;*cap*^*f*^

a *sdr loci are composed of genes sdrB, sdrC and sdrD*.

b *ica locus encoding polysacharide intercellular adhesin (PIA)*.

c *fibronectin binding protein A gene (fbpA)*.

d *biofilm-associated gene (veg)*.

e *hemolysin III family gene (hly-III)*.

f *capsule gene clusters (cap)*.

g *putative gene for adenosine synthase A (adsA)*.

*N/A, not analyzed*.

–*, not detected*.

**Figure 2:**
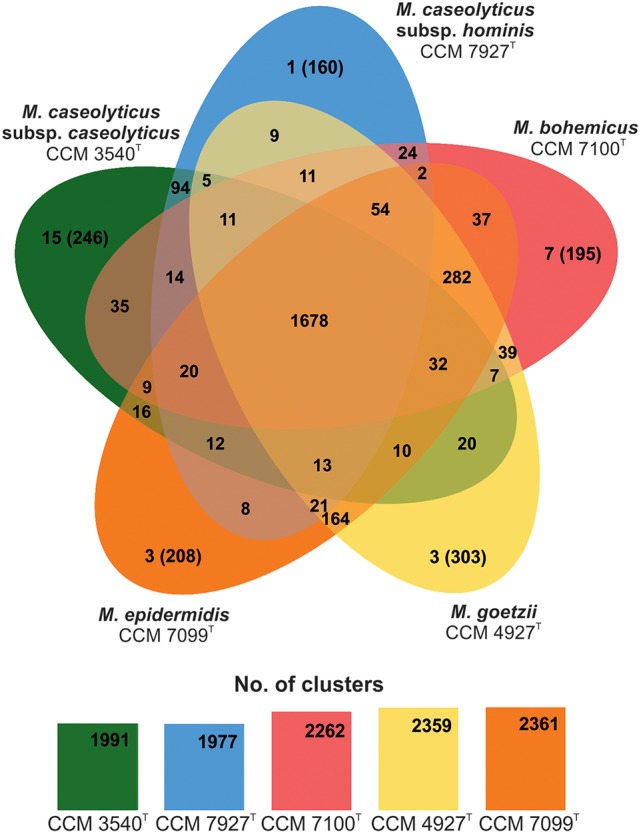
Venn diagram showing shared orthologous protein clusters among genomes of human macrococcal isolates and *Macrococcus caseolyticus* subsp. *caseolyticus* subsp. nov. CCM 3540^T^. The number of protein clusters predicted by RAST comprised of multiple protein families is indicated for each genome. The orthologous clusters were identified with the default parameters, 1e^−5^ e-value cutoff for all protein similarity comparisons and 1.5 inflation value for the generation of orthologous clusters. The number of singletons specific for each genome is shown in brackets.

Examination of the macrococcal pan-genome showed that five macrococcal representatives (CCM 3540^T^, CCM 4927^T^, CCM 7927^T^, CCM 7099^T^, and CCM 7100^T^) share 1678 orthologous protein clusters (Figure 2). On a pairwise basis, the type strain *M. caseolyticus* subsp. *caseolyticus* subsp. nov. CCM 3540^T^ shares additional 94 distinctive orthologous groups with *M. caseolyticus* subsp. *hominis* subsp. nov. CCM 7927^T^. All group 2 strains share 282 orthologous groups, which is the largest common gene pool in the analyzed sample. Strain CCM 4927^T^ shared the most with CCM 7099^T^, with 164 orthologous groups. Hence these results support the outcomes of the phylogenetic analysis and shows that CCM 4927^T^ and CCM 7099^T^ are more related to each other than to strain CCM 7100^T^. According to the Gene Ontology (GO) enrichment analysis among the three group 2 strains, five enriched pathways were found: quinone binding, ATP synthesis-coupled electron transport, NADH dehydrogenase (ubiquinone) activity, calcium ion transmembrane transport, and respiratory chain complex, and strains CCM 7099^T^ and CCM 4927^T^ had an over-represented lipid metabolic process.

**Figure 3:**
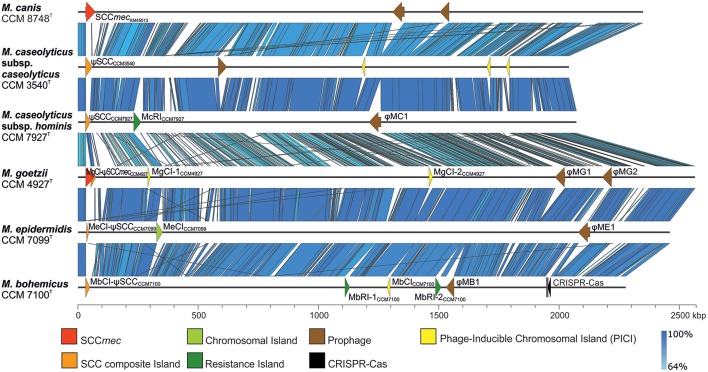
Whole-genome comparison of human macrococci and *Macrococcus caseolyticus* subsp. *caseolyticus* subsp. nov. CCM 3540^T^ and *Macrococcus canis* CCM 8748^T^ (GenBank accession number CP021059) with highlighted variable genetic elements. Variable genetic elements are color-coded according to the legend.

**Figure 4:**
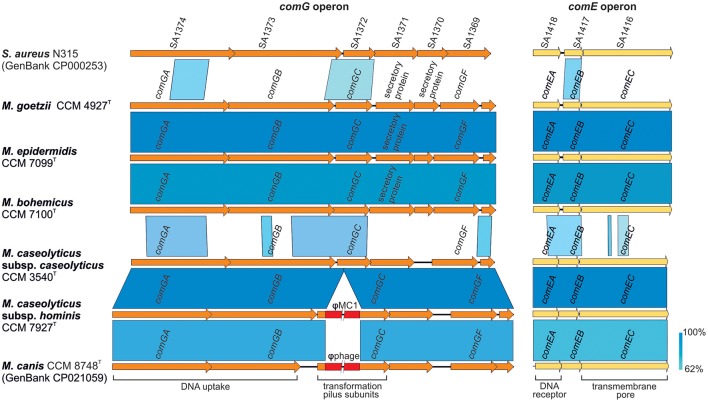
Genetic structure of *com* operon in human macrococci. The picture shows a comparison of the *comG* and *comE* operons, which might be functional in macrococcal strains CCM 4927^T^, CCM 7099^T^, and CCM 7100^T^. The genes are annotated according to Fagerlund et al. (2014); ComGA is a traffic ATPase, ComGB a polytopic membrane protein and ComGC major component of a pilus ensuring the competence of bacterial cells during transformation. Genes *comGD, comGE*, and *comGF* encodes minor pilin structure. ComEA is a membrane DNA receptor, ComEB a late competence protein, and ComEC a channel for DNA uptake.

Comparative genomics of human macrococci discovered huge genome structure plasticity, which might give them an advantage in terms of survival in diverse environments (Figure 3). We hypothesize that such genomic variability, particularly in group 2 strains, is attributed to the presence of *com* operons. These operons play a key role in the uptake of exogenous DNA during the transformation of *Streptococcus pneumoniae* (Muschiol et al., 2015) and *Bacillus subtilis* (Chung and Dubnau, 1995) and possibly sustain functionality in macrococci. All sequenced human *Macrococcus* isolates have been found to contain part of the putative *comG* operon (*comGA-comGC*), the complete *comE* (*comEA-comEC*) operon, and the *dprA* gene, which is another key factor in transformations and mediates DNA recombination. This suggests that transformation may lead to the extensive genomic variability and larger genome size of group 2 macrococci isolates. Conversely the negative lysogenic conversion of the *comG* locus caused by the presence of prophage ϕMC1 in strain CCM 7927^T^ from group 1 strains could be responsible for the low accessory genome variability in *M. caseolyticus* subsp. *hominis* subsp. nov. (Figure 4).

The presence of a CRISPR-Cas system and restriction-modification (R-M) systems in macrococcal genomes corresponds with the need to limit the burden of foreign DNA. Strain CCM 7927^T^ carries a Type II R-M system with methyltransferase and the endonuclease from the *Dpn*II family. Strains CCM 4927^T^, CCM 7099^T^ and CCM 7100^T^ contain Type I R-M systems consisting of three genes (*hsdM, hsdS*, and *hsdR*).

Accessory genes that are not essential in all bacterial strains were identified in the human macrococcal genomes. The highest number of genome-specific proteins, with 303 singletons, was found in the CCM 4927^T^ genome followed by CCM 3540^T^, CCM 7099^T^, CCM 7100${}_{,}^{\text{T}}$ and CCM 7927^T^ with 246, 208, 195, and 160 singletons, respectively. While accessory genes localized outside MGEs constitute 17% of the predicted coding sequences of *M. caseolyticus* CCM 3540^T^ and CCM 7927^T^, for the strains CCM 4927^T^ and CCM 7099^T^ the accessory genes localized outside MGEs constitute 31–32%, and for strain CCM 7100^T^ 24%. The higher percentage of accessory genes in strains CCM 4927^T^ and CCM 7099^T^ is consistent with their expected ability to receive foreign DNA via transformation, in contrast to strain *M. caseolyticus* subsp. *hominis* subsp. nov. CCM 7927^T^, where negative lysogenic conversion of the *comG* locus was identified. Although negative lysogenic conversion was not observed in strain CCM 7100^T^, the lower proportion of accessory genes outside MGEs might be caused by the presence of the complete CRISPR-Cas system.

### New variant of type II-C CRISPR-cas system

Clustered, regularly interspaced, short, palindromic repeats (CRISPR) loci, together with their CRISPR-associated (Cas) proteins provide the bacteria with adaptive immunity against the invasion of bacteriophages, plasmids, and other MGE. The CRISPR-Cas system found in *M. bohemicus* sp. nov. CCM 7100^T^ is demarked by two 36-bp anti-repeats (GAAGCCATTCGCTTCATTCTAAAAGATCATAGTTAA; TCATAGTTCTAAAACACTCGTGCAATCCTACTCTTA). The CRISPR locus is 5,888 bp long, and contains 90 spacers separated by 36-bp-long repetitive sequences (GTTTCACTTCATTCTAAAAGATCATAGTTCTAAAAC). Genes for Cas proteins were found neighboring the CRISPR arrays and were categorized as Cas1, Cas2 and Cas9. Cas1 exhibited 60% amino acid identity with the type II CRISPR-associated endonuclease Cas1 of *S. pseudintermedius* (Ben Zakour et al., 2011); and 57% amino acid identity with the type II CRISPR-associated endonuclease Cas1 of *Staphylococcus simulans* (GenBank accession no. CP023497). Cas2 exhibits 72% amino acid identity with the CRISPR-associated endonuclease Cas2 of *Staphylococcus delphini* (Verstappen et al., 2017). The whole CRISPR-Cas locus exhibited high nucleotide identity (68%) to the type II-C CRISPR-Cas of *S. simulans* strain FDAARGOS_383 (GenBank accession no. CP023497), hence this region was classified as CRISPR-Cas type II-C.

### Virulence factors

The pathogenic potential of bacteria depends on the production of virulence factors and the presence of antimicrobial resistance determinants. Human macrococci, as well as closely related coagulase-negative staphylococci, seem to be a potential reservoir of virulence and resistance genes for staphylococci (Becker et al., 2014). Putative genes carrying the motifs associated with bacterial virulence factors were predicted in analyzed genomes using the virulence factor database (VFDB) (Table 2). The locus encoding polysaccharide intercellular adhesin (PIA) was identified in strains CCM 4927^T^, CCM 7099^T^, and CCM 7100^T^ from group 2. PIA has been described as a factor involved in staphylococcal biofilm accumulation (Rohde et al., 2010). Highly heterogenic gene homolog of biofilm synthesis protein poly-beta-1,6-N-acetyl-D-glucosamine synthase (PNAG) have been identified not only across the genus *Staphylococcus*, but also in Gram-negative human pathogens. A putative macrococcal PNAG synthase gene homolog shares the highest amino acid identity (64%) with a protein of *Bacillus onubensis* (Dominguez-Moñino et al., 2018).

The biofilm-associated *veg* gene for a small conserved Veg protein (87 aa) is present in all sequenced macrococcal genomes. Veg protein is conserved among the four sequenced strains and BLAST analysis showed the *veg* gene to be conserved not only among the whole genus *Macrococcus* but also in coagulase-negative staphylococci and other low-G+C Gram-positive bacteria from the phylum *Firmicutes*. The function of the Veg coding gene has been studied in *B. subtillis*, where the transcription of *veg* was increased during exponential growth and sporulation, supporting its function as an additional regulatory protein contributing to the control of matrix production (Lei et al., 2013).

In all sequenced human macrococcal genomes, the putative gene *fbpA* encoding a potential virulence factor, fibronectin binding protein A (FbpA), was identified. This protein is highly conserved across the genus *Macrococcus* with more than 74% amino acid identity. The FbpA protein of strain CCM 4927^T^ shares 52% amino acid identity and 98% query coverage with the FbpA of *Staphylococcus saprophyticus* (GenBank accession no. WP_061854885) (Mukherjee et al., 2016). The mutual nucleotide identity of the *fbpA* of the strain CCM 4927^T^ compared to strain CCM 7099^T^ and CCM 7100^T^ is 79% and compared to CCM 7927^T^ is 73%. FbpA is one of the most important adhesins for binding to human fibronectin. Human fibronectin participates in eukaryotic cellular processes, such as adhesion, migration and differentiation, and FbpA is important during staphylococcal and streptococcal infection, since it allows the pathogens to bind to host epithelial cells, facilitates their internalization (Joh et al., 1999) and systemic spread within the host (Christie et al., 2002).

**Figure 5:**
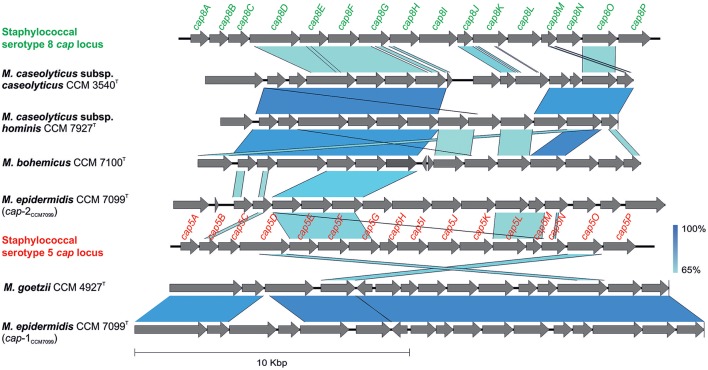
Comparison of genetic structure of putative capsule (*cap*) gene clusters in sequenced genomes of human macrococci with staphylococcal *cap5* (GenBank accession number U81973) and *cap8* (GenBank accession number U73374) loci.

The genome analyses revealed putative capsule gene clusters in all sequenced genomes, composed of putative *cap* genes, which are arranged consecutively. Moreover, strain CCM 7099^T^ contains two *cap* loci (cap-1_CCM7099_, cap-2_CCM7099_) in its chromosome. Many genes involved in the biosynthesis of the capsule are similar among the members of the genera *Staphylococcus, Streptococcus*, and *Bacillus*, with similar capsule gene clusters within the genus *Macrococcus*. The *cap* locus is involved in the expression of the capsule and enhances virulence by phagocytosis evasion. Most staphylococci express the serotype 5 or 8 capsular polysaccharides (Watts et al., 2005). The staphylococcal *cap5* and *cap8* loci are comprised of an ~17.5 kb region with 16 closely linked genes (O'Riordan and Lee, 2004), which is comparable to the genetic structure of the 15 – 18 kb macrococcal *cap* region with 13 to 17 ORFs (Figure 5). The highest similarity (93% nucleotide identity, coverage 89%) of the *cap* locus was observed between cap-1_CCM7099_ and cap_CCM4927_ from CCM 4927^T^. Both regions exhibit partial homology to the staphylococcal serotype 5 *cap* genes (Sau et al., 1997), whereas the cap-2_CCM7099_ and cap_CCM7100_ from CCM 7100^T^, and cap_CCM7927_ from CCM 7927^T^ are homologous to the staphylococcal serotype 8 *cap* genes (Sau and Lee, 1996).

A key aspect of bacterial virulence lies in the ability to target the host cell membrane with many membrane-damaging toxins and peptides. Another putative virulence factor found in all human *Macrococcus* genomes is the predicted membrane channel-forming protein YqfA from the haemolysin III family (*hly*-III). Virulence factor *hly*-III has been described in *Bacillus cereus* to cause erythrocyte lysis as a result of the formation of transmembrane pores (Baida and Kuzmin, 1996). In the multiple sequence alignment of the macrococcal *hly-III* genes, two clusters corresponding to phylogenetic group 1 and group 2 were distinguished. However, the haemolysis determined on Columbia blood agar supplemented with sheep blood was not observed for human macrococci. Further analysis of the *hly* genes found several single-nucleotide polymorphisms that might result in the expression of non-functional haemolysin.

The putative gene for adenosine synthase A (AdsA), which is a critical virulence factor in staphylococci, was found in strains CCM 4927^T^ and CCM 7099^T^. AdsA is a cell wall-anchored enzyme that converts adenosine monophosphate to adenosine (Thammavongsa et al., 2009). Kim et al. (2012) showed that the staphylococcal synthesis of adenosine enabled by AdsA allows the pathogen to escape from phagocytic clearance in blood, to form organ abscesses, and to block host adaptive immune defense mechanisms. AdsA from strain CCM 4927^T^ exhibited 59% amino acid identity and 74% coverage with that described in *S. aureus* (Thammavongsa et al., 2011). The mutual similarity of AdsA from macrococcal strains CCM 7099^T^ and CCM 7100^T^ is 95%.

### *OriC* environ and staphylococcal cassette chromosome-like elements

A comparison of the sequenced *Macrococcus* genomes revealed a high heterogeneity in the region near 3′ *oriC*. This region was found to be distinctive within the genus *Staphylococcus* (Takeuchi et al., 2005) and correspondingly macrococcal genomes are highly diverse in this region (Figure 3). All human macrococcal isolates carry a pseudo *Staphylococcus* chromosome cassette (ψSCC) inserted into the *rlmH* (*orfX*) gene in the vicinity of the *oriC*. Moreover β-lactam susceptibility testing revealed that strain CCM 4927^T^ is resistant to β-lactam antibiotics, including oxacillin, thus pointing to the presence of a *mecB* gene, which was indeed identified as part of ψSCC*mec*_CCM4927_. The *mec*B gene of macrococcal origin was recently detected on a plasmid in MRSA strain UKM4229 (Becker et al., 2018), emphasizing that macrococcal resistance genes could be transferred to staphylococci, especially when the two species share the same habitat.

**Figure 6:**
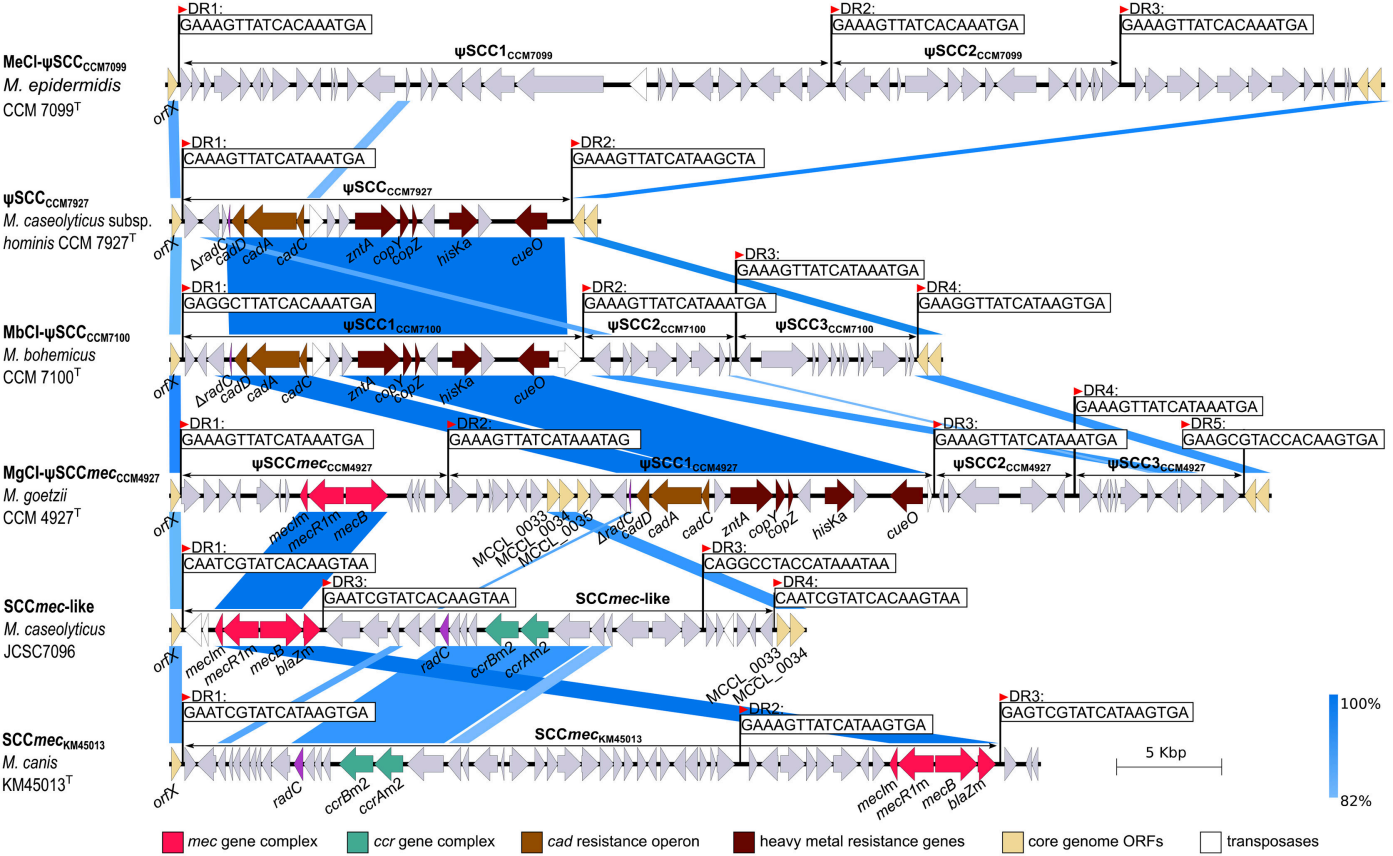
Description and structural comparison of staphylococcal cassette chromosome (SCC)-like elements localized to *oriC* environs of *Macrococcus caseolyticus* subsp. *hominis* subsp. nov. CCM 7927^T^, *Macrococcus epidermidis* sp. nov. CCM 7099^T^, *Macrococcus bohemicus* sp. nov. CCM 7100^T^, and *Macrococcus goetzii* sp. nov. CCM 4927^T^ with SCC*mec* elements in *Macrococcus canis* CCM 8748^T^ (GenBank accession number HG970732) and *Macrococcus caseolyticus* JCSC7096 (GenBank accession number AB498756). Direct repeats (DR) demarking the SCCs and related elements are shown. Arrows indicate ORFs and their orientation in the chromosome.

ψSCC*mec*_CCM4927_ is 12,779 bp long, bordered by imperfect 18-bp direct repeats. This element harbors 14 ORFs (Figure 6), including the *mecB* gene, which shares 98% nucleotide identity with other *mecB* genes of sequenced macrococci (Gómez-Sanz et al., 2015). The regulators of the *mecB* transcription, the repressor *mecI* and inducer *mecR1*, were also present within the *mec* gene complex, but this locus as well as the whole genome, did not contain the *blaZ* gene, which is associated with the class E *mec* complex in other macrococci (Tsubakishita et al., 2010). Hence the structure of the complex could be the missing link between the antecedent form of the class E *mec* complex (*mecI*–*mecR*–*mecA*–*blaZ*) and the descendent class A *mec* complex (*mecI*–*mecR*–*mecA*) present in SCC*mec* type II, III and VIII, as discussed by Tsubakishita et al. (2010). No cassette chromosome recombinase (*ccr*) genes or their homologs were found in ψSCC*mec*_CCM4927_, neither transposase genes were present, so it is uncertain whether this element alone could be transferred.

Three additional copies of the insertion sequence site (ISS) were found in the genomic sequence of strain CCM 4927^T^ that form the boundaries of three adjacent ψSCC elements forming a composite island designated MgCI-ψSCC*mec*_CCM4927_ composed of ψSCC*mec*_CCM4927_, ψSCC1_CCM4927_, ψSCC2_CCM4927_, and ψSCC3_CCM4927_.

ψSCC1_CCM4927_ is 23,281 bp long, carries 24 ORFs and consists of two separate parts. The first part contains hypothetical genes from which three ORFs exhibit 92% sequence identity to genes previously identified as *M. caseolyticus* JCSC5402 chromosomal genes MCCL_0033, MCCL_0034, and MCCL_0035 (Baba et al., 2009). The second part contains a cluster of heavy metal resistance genes with a *cad* operon (*cadDAC* genes), *zntA* gene, *cop* operon (*copYZ*), histidine kinase *hisK* gene and multicopper oxidase *cueO*. This cluster has a G+C content of 35.86 mol%, which is considerably higher than the average G+C content of the rest of the MgCI-ψSCC_CCM4927_ and *Macrococcus* genome. Based on the gene composition and higher G+C content, we propose that this heavy metal resistance gene cluster is a transposon; it was also found in the ψSCCs of strains CCM 7100^T^ and CCM 7927^T^. This transposon was probably integrated into the genome independently before the creation of the ψSCC1_CCM4927_ element, which strengthens the hypothesis that SCCs are prone to the accumulation of MGE and dynamic evolution (Indráková et al., 2016). ψSCC2_CCM4927_ is a 6,682-bp element carrying the gene for an additional threonine-tRNA-ligase and four genes coding for hypothetical proteins. ψSCC3_CCM4927_ is 7,899 bp in size and it contains 11 ORFs, comprised of putative isochorismatase, a 3-ketoacyl reductase paralog, and hypothetical proteins.

During the *oriC* environ analysis of the strain CCM 7100^T^, a composite island MbCI-ψSCC_CCM7100_ of three ψSCCs (ψSCC1_CCM7100_, ψSCC2_CCM7100_, and ψSCC3_CCM7100_) was identified (Figure 6). ψSCC1_CCM7100_ has a length of 19,392 bp and encodes 20 ORFs. Apart from two hypothetical proteins and putative histone acetyltransferase, the ψSCC1_CCM7100_ element includes a heavy metal resistance transposon comparable to the one found in CCM 4927^T^. The second element inserted into the *rlmH* gene of CCM 7100^T^ is 7,162-bp-long ψSCC2_CCM7100_, comprised of 8 ORFs, namely the putative transcriptional regulator from the XRE family, putative HNH endonuclease, and hypothetical proteins. The third element in the region is ψSCC3_CCM7100_, which is 8,501 bp long with 12 ORFs coding for daunorubicin resistance protein, putative dehydrogenases, putative arsenic efflux pump protein ArsB and arsenic resistance operon repressor protein ArsR, and several hypothetical proteins.

The chromosome of strain CCM 7927^T^ contained one ψSCC similar to ψSCC1_CCM7100_ (Figure 6). The 18,494-bp-long ψSCC_CCM7927_ carries 20 ORFs and an almost identical transposon to the one detected in strain CCM 7100^T^.

Strain CCM7099^T^ also carries a composite island inserted into the *rlmH* gene. The island is made of two ψSCCs, 31.0-kb-long ψSCC1_CCM7099_ and 13.8-kb-long ψSCC2_CCM7099_. These two cassettes are not similar in their gene composition to any of the above-described ψSCCs (Figure 6), and they code for transcriptional regulators, putative resistance (blasticicdin S deaminase, bleomycin resistance protein, aminoglycoside transferase) and virulence factors (sortase and LPXTG motif containing protein), and other uncharacterized proteins.

### Transposons harboring heavy metal resistance genes

The transposon found in MgCI-ψSCC*mec*_CCM4927_ of strain CCM 4927^T^ and also within the ψSCCs of strains CCM 7100^T^ and CCM 7927^T^ is presumably inserted into the *radC* gene, whose remnants were found directly upstream of the transposon sequence (Figure 6). Notably, other transposons of *Firmicutes* exhibit a strong preference for the insertion site within *radC* (Müller et al., 2013). The *cadDAC* operon was followed by the transposase from the IS*30* family (98% amino acid identity to Tnps from *Macrococcus, Enterococcus*, and *Staphylococcus*). A similar *cad* resistance region was found in *S. pseudintermedius* (Chanchaithong et al., 2016). Inverted repeats delimiting the transposon or the second part of the *radC* gene were not found, so the boundary on the right hand-side was determined by the presence of the transposase from the IS*4* family in the CCM 7100^T^ chromosome (66% amino acid identity to Tnp from *Ureibacillus thermosphaericus* and *Lysinibacillus macroides*) and given the presence of two different transposases genes, it is thus possible that the macrococcal transposon is concatenated from two separate transposons that confer heavy metal resistance.

Apart from the transposons found in the *oriC* environ of CCM 4927^T^, CCM 7100^T^, and CCM 7927^T^, another transposon conferring resistance to heavy metals was identified in the genomes of the three above-mentioned human *Macrococcus* strains. The 7.4-kb-long transposon is bordered by insertion sequences with two transposase genes from the IS*3* family and apart from lead-, cadmium- zinc- and mercury/heavy metal-transporting ATPase genes, also contains a resolvase gene and an ArsR transcriptional regulator gene. Inverted repeats in the detected transposon are almost identical to a 123-bp-long sequence in IS*1216E* from *Enterococcus faecium* (Arthur et al., 1997).

### Other chromosomal and resistance islands

The resistance island designated McRI_CCM7927_ encoding macrolide resistance and harboring a putative gene for chaperone protein ClpB, which is part of a stress-induced multi-chaperone system, was found in strain CCM 7927^T^ (Figure 3). McRI_CCM7927_ is integrated into the 3' end of the 30S ribosomal protein S9 gene (*rpsI*) localized upstream of the gene for a hypothetical protein of AAA family ATPase, which is the same locus as the one used by McRI_mecD_ carrying the *mecD* gene in two *M. caseolyticus* strains described earlier by Schwendener et al. (2017). McRI_CCM7927_ carries 22 ORFs, including an integrase. There is a completely different chromosomal island (MeCI_CCM7099_) in the genome of CCM 7099^T^, sharing only the same *att* site and integrase (94% nucleotide similarity) with McRI_CCM7927_ (Figure 3). MeCI_CCM7099_ does not contain any known resistance or virulence genes. No chromosomal island was integrated into the *rspI* gene of CCM 7100^T^, and even the gene for AAA family ATPase was missing.

Strain CCM 7100^T^ carries two genomic islands, designated MbRI-1_CCM7100_ and MbRI-2_CCM7100_. MbRI-1_CCM7100_ is ~19 kb long and carries genes for resistance to heavy metals and copper. This genomic island is integrated into one of the two copies of the gene for LSU ribosomal protein L33p. MbRI-2_CCM7100_ is cca 23 kb long and integrated into the *radC* homolog encoding a DNA repair protein (Figure 3). This island harbors the *optrA* gene cluster encoding oxazolidinone/phenicol resistance, which was described in *S. sciuri* (Li et al., 2016), and type I R-M system.

### Phage-induced chromosomal islands

MGEs designated phage-inducible chromosomal islands (PICIs) that can contribute to host adaptation and virulence (Penadés and Christie, 2015) were identified in genomes of strains CCM 4927^T^ and CCM 7100^T^ (Figure 3). Macrococcal PICIs have a typical modular structure that facilitates their integration, replication and excision such as *S. aureus* pathogenicity islands (SaPIs), the prototype members of these elements. Strain CCM 4927^T^ has two putative PICIs in the genome. The 11-kb-long island MgCI-1_CCM4927_ is integrated between the genes for hypothetical L-lactate dehydrogenase subunit YkgG and phospatidylethanolamine N-methyltransferase gene. The 22-bp-long *att* site of MgCI-1_CCM4927_ is TTGTTTTAGATGATAAATAATA. Apart from many hypothetical genes, no virulence or resistance gene homologs were identified. The second 15-kb-long island MgCI-2_CCM4927_ is integrated between the genes for chorismate synthase and nucleoside diphosphate kinase. The 24-bp *att* site of MgCI-2_CCM4927_ is TGCCCTTTTTCTGCCCTTTTTTTA, which is the same *att* site as for the 15-kb-long island MbCI_CCM7100_ located in the genome of strain CCM 7100^T^. Although both islands share the integration site and their integrases have 74% mutual amino acid identity, their nucleotide sequences are only similar in 8% of their total length. There is a gene encoding a plasmid antitoxin from the HigA proteins family at the end of the island MbCI_CCM7100_. The toxin associated with this antitoxin gene was identified on the smaller plasmid pZKMS1 from strain CCM 7100^T^.

### Prophages

**Figure 7:**
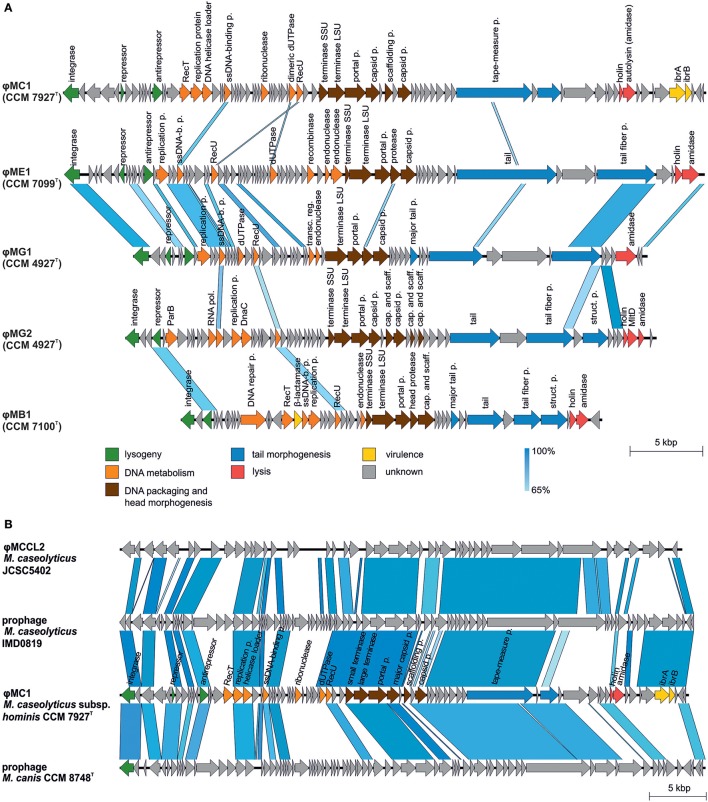
Comparative genomics of macrococcal bacteriophages. **(A)** Mutual comparison of highly divergent prophage genomes from human macrococci: φMC1 (CCM 7927^T^), φME1 (CCM 7099^T^), φMG1 (CCM 4927^T^), φMG2 (CCM 4927^T^), and φMB1 (CCM 7100^T^). **(B)** Genome comparison of converting phage φMC1 from *Macrococcus caseolyticus* subsp. *hominis* subsp. nov. CCM 7927^T^ with *M. caseolyticus* JCSC5402 phage φMCCL2, phage from *M. caseolyticus* IMD081D and phage from *M. canis* CCM 8748^T^. Genomes were aligned using the blastn algorithm and similar regions with more than 65% identity are indicated. The positions and orientations of the coding regions are represented by arrows. Genome modules are color-coded according to the legend.

Prophages were identified in all sequenced genomes of *Macrococcus* spp. strains (Table 3). The detected prophages in human macrococci exhibit low similarities to any known phage or putative prophage in the databases except for ϕMC1 (CCM 7927^T^). Also, a comparison of all five prophages identified in human macrococci uncovered a low level of synteny, only the prophages ϕMG1 (CCM 4927^T^) and ϕME1 (CCM 7099^T^) exhibit 20% nucleotide identity and have the same integration site in their host genomes (Figure 7A). Although the prophages have unique gene compositions, they follow the typical siphoviral modular structure (Kahánková et al., 2010).

**Table 3:** Prophages identified in genomes of human macrococci and their characteristics.

**Strain**	**Phage name**	**Size (bp)**	**G+C (mol%)**	**ORFs**	**Integration site**	***att* sequence**	**Similar prophages**	
							**Host**	**GenBank accession no**.
CCM 7927^T^	ϕMC1	50167	34.72	69	*comGC*	CATTTCAATTAAAGT	*M. caseolyticus* JCSC5402	AP009484
CCM 4927^T^	ϕMG1	39954	32.83	58	between *TsaD* and *ABC transporter*	CGAACACATGTACTTGTACACAA	*Staphylococcus agnetis* 908	CP009623
	ϕMG2	41282	34.10	60	*tRNA-*Leu	ACCCCGACCGGTGGTACTA	*M. goetzii* sp. nov. CCM 4927 (ϕMG1)	this study
CCM 7099^T^	ϕME1	49155	32.71	73	between *TsaD* and *ABC transporter*	TTGTGTACAAGTACATGTGTTCGCGTGGATAA	*M. caseolyticus* IMD0819	CP021058
CCM 7100^T^	ϕMB1	32855	33.87	47	tRNA-Arg	TTAGCGTCCTGGGAGG	*M. caseolyticus* IMD0819	CP021058

Comparative analysis showed that the phage ϕMC1 is highly similar in its major genomic modules, including its integrase and integration site, to the prophages of *M. caseolyticus* IMD0819 (Schwendener et al., 2017) and *M. canis* CCM 8748^T^ (Gobeli Brawand et al., 2018). ϕMC1 also exhibits high similarity to the φMCCL2 of *M. caseolyticus* JCSC5402 (Baba et al., 2009) (Figure 7B). An important feature of ϕMC1 is its suspected lysogenic conversion. The phage ϕMC1 is integrated into the *comGC* gene of the *comG* operon, thus causing its negative conversion (Figure 4). Two putative immunoglobulin-binding regulators IbrA and IbrB are encoded by the phage genome. IbrA and IbrB are hypothesized to be possible virulence factors in *Escherichia coli* (Sandt et al., 2002) with 33% amino acid and 50% amino acid similarity, respectively, to those harbored in the ϕMC1 genome. These regulators activate genes for immunoglobulin-binding proteins (Eib) (Rubin et al., 2017). Genes encoding IbrA and IbrB were previously found in the prophages of different distantly related bacterial species (Castillo et al., 2014; Laanto et al., 2015).

**Figure 8:**
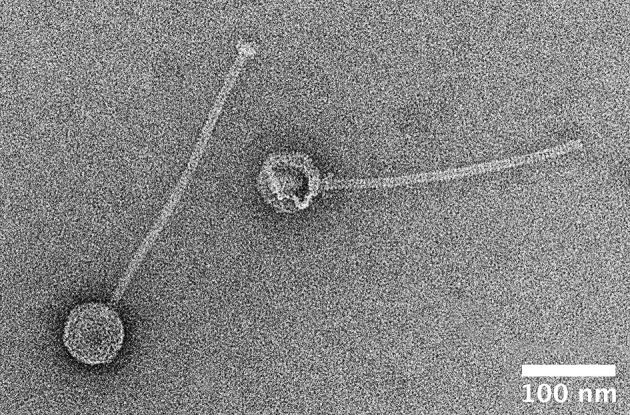
Transmission electron microscopy image of negatively stained particle of phage φMC1 induced from *Macrococcus caseolyticus* subsp. *hominis* subsp. nov. CCM 7927^T^ by UV-light.

Phages from all the strains were induced by UV irradiation. The lysate of *M. caseolyticus* subsp. *hominis* subsp. nov. CCM 7927^T^ contained phage particles detectable by TEM. Morphological analysis by TEM with negative staining revealed that this bacteriophage, designated ϕMC1 (vB_McaS_7927), belongs to the *Siphoviridae* family. The phage particles consist of an icosahedral head (B1 morphology) with a flexible, non-contractile tail ending with a base plate. The diameters of the phage head are from 66.5 to 69.5 nm, and tail length is ~310 nm (Figure 8). The structural proteins of the ϕMC1 phage particles separated by SDS-PAGE and analyzed by mass spectrometry matched a prophage sequence identified in the genome of the monolysogenic host strain CCM 7927^T^.

### Plasmids

Extrachromosomal plasmids were identified in all sequenced genomes of human macrococci (Table 2). All plasmids contain many hypothetical genes without any homologs in the databases.

The 3,842 bp-long plasmid pZKMH1 was identified in the genome of CCM 7927^T^. This plasmid carries a transposable element with a small multidrug resistance (*smr*) cassette-like structure, usually found in small non-conjugative plasmids, such as staphylococcal pSK41 (Costa et al., 2013). The cassette encodes two Smr proteins conferring resistance to quaternary cation compounds and an associated transcription factor from the ribbon–helix–helix superfamily (Schreiter and Drennan, 2007; Bay and Turner, 2012). These genes could serve as a defense mechanism against active oxygen toxicity and could prevent the oxidative damage of DNA (Demple et al., 1983). The same *smr* cassette-like structure was also identified in the chromosomes of strain CCM 7100^T^ and newly described species *Auricoccus indicus*, which is distantly related to macrococci (Prakash et al., 2017), hence pointing to the intergeneric horizontal transfer of these resistance genes.

34,802 bp-long pZKMB1 and 69,509 bp-long pZKMB2, low-copy number plasmids from strain CCM 7100^T^, encode putative *tra* genes, suggesting their conjugative transfer. Nevertheless, only pZKMB1 harbors other genes necessary for conjugation. The conjugative potential of these plasmids has not been tested due to the absence of suitable selection markers. The conjugation-essential nick region 5′GTGTGTAAGTGCG↓CCCTTG3′ that matches the consensus sequence 5′NcgtNtaAgtGCGCcCTta 3′for the RSF10-*ori*T (Grohmann et al., 2003) was identified in the plasmid pZKMB1. The MbA/MobL superfamily domain of the putative DNA relaxase (TraA) of pZKMB1 possesses two out of three motifs that are typically found in all conjugative DNA relaxases in Gram-positive bacteria (Zechner et al., 2017); motif I with a conserved Tyr residue binds the DNA backbone, and motif III with two His residues is involved in nucleophilic attack at the nick region.

The plasmid pZKMB2 shares short regions with 73% nucleotide identity with the pMCCL2 plasmid from *M. caseolyticus* strain JCSC5402. Unlike pMCCL2 that carries an unusual form of the *mec* gene complex (*mecB-mecR1*_m_-*mecI*_m_-*blaZ*_m_) (Tsubakishita et al., 2010), the pZKMB2 plasmid encodes the putative stage V sporulation protein B (SpoVB), responsible for the late stage of spore development (Vasudevan et al., 2009). In strain CCM 7099^T^, the 11,867 bp-long plasmid pZKME1 encoding 12 ORFs with unknown function was identified. The small cryptic 1,913-bp-long plasmid pZKMG1 was identified in strain CCM 4927^T^. pZKMG1 only encodes a replication protein with 94% amino acid identity to the replication protein of pSTH6 from *S. saprophyticus* (Heir et al., 1998).

### Species description protologues

Protologues describing the four aforementioned novel taxa are given below. Phenotypic tests distinguishing the novel macrococcal taxa from the phylogenetically close species *M. canis* and *M. caseolyticus* are presented in Table 4. The ability to use carbon sources via respiration, determined in Biolog GP2 MicroPlate assays is listed in Table S2.

**Table 4:** Phenotypic characteristics that differentiate novel macrococci from phenotypically related *Macrococcus caseolyticus* subsp. *caseolyticus* subsp. nov. and *Macrococcus canis*.

**Test**	1*	2*	**3**	**4**	**5**	**6**
Growth at 10°C	+	–	–	+	+	+
Growth at 45°C	+	+	–	+	–	–
Growth in thioglycolate	+	–	–	w	w	+
Yellow pigmented	–	+	–	–	–	–
Chymotrypsin	+	+	w	+	–	+
Acid from: ribose	+	+	–	–	–	–
galactose	+	–	–	+	+	+
mannitol	–	+	+	–	–	–
lactose	+	–	–	+	+	+
salicine	–	–	–	+	+	+
turanose	–	–	–	+	+	+
L-arabinose	–	–	–	+	–	–
melezitose	–	–	–	+	+	–

*Strains: 1. M. caseolyticus subsp. caseolyticus subsp. nov. CCM 3540^T^; 2. M. caseolyticus subsp. hominis subsp. nov. CCM 7927^T^; 3. M. canis CCM 8748^T^; 4. M. goetzii sp. nov. CCM 4927^T^; 5. M. epidermidis sp. nov. CCM 7099^T^; 6. M. bohemicus sp. nov. CCM 7100^T^*.

+, positive; w, weak positive; –, negative;

* data valid for type strain as well as subspecies description; all data of type strains were taken from this study

### Description of *macrococcus caseolyticus* subsp. *hominis* subsp. nov.

*Macrococcus caseolyticus* subsp. *hominis* subsp. nov. (ho′mi.nis L. gen. n. *hominis* of man, indicating that the strains were isolated from humans).

Cells are Gram-stain positive spherical cocci, occurring predominantly in pairs and clusters, non-spore forming and non-motile. Colonies on TSA agar are circular, whole margin, flat, smooth, shiny, 1–2 mm in diameter, aerobic and pigmented a yellowish orange. No haemolytic activity. Grows in the presence of up to 7.5% NaCl, at 15°C (weak) and 48°C but not at 10°C or 50°C. No growth in the presence of 10% or more NaCl and in a thioglycolate medium. Catalase, oxidase, pyrrolidonyl arylamidase, nitrate reduction and Voges-Proskauer test (acetoin) positive. Weak hydrolysis of gelatin. Coagulase, clumping factor, urease, arginine dihydrolase, and ornithine decarboxylase negative. Susceptible to furazolidon (100 μg) and resistant to novobiocin (5 μg) and bacitracin (10 IU). Hydrolysis of esculin and Tween 80 negative. Acid phosphatase, alkaline phosphatase (weak), esterase (C4), esterase lipase (C8), chymotrypsin (weak) and naphthol-AS-Bi-phosphohydrolase positive. Lipase (C14), leucine arylamidase, valine arylamidase, cystine arylamidase, trypsin, α-galactosidase, β-galactosidase, β-glucosidase, N-acetyl-β-glucosaminidase, α-mannosidase and α-fucosidase negative. Acid is produced from glycerol, ribose, D-glucose, D-fructose, mannitol, maltose, sucrose and trehalose. Acid is not produced from erythritol, D-arabinose, L-arabinose, D-xylose, L-xylose, adonitol, β-methyl-D-xyloside, galactose, mannose, sorbose, rhamnose, dulcitol, inositol, sorbitol, α-methyl-D-mannoside, α-methyl-D-glucoside, N-acetyl glucosamine, amygdalin, arbutin, salicin, cellobiose, lactose, melibiose, inulin, melezitose, D-raffinose, starch, glycogen, xylitol, β-gentiobiose, D-turanose, D-lyxose, D-tagatose, D-fucose, L-fucose, D-arabitol, L-arabitol, gluconate, 2 keto-gluconate and 5 keto-gluconate. Variable biochemical reactions were obtained for the hydrolysis of DNA (1 of 4 positive), β-glucuronidase (2 of 4) and α-glucosidase (2 of 4). Susceptible to ampicillin, cefoxitin, cephalothin, ciprofloxacin, clindamycin, gentamicin, chloramphenicol, imipenem, kanamycin, neomycin, oxacillin, penicillin G, sulphamethox/trimethoprim (cotrimoxazol), tetracycline and vancomycin. Resistant to erythromycin. The DNA G+C content of the type strain is 36.79 mol% (calculated from WGS). The peptidoglycan type is A3α Lys-Gly_3_-Ser. The major fatty acids are C_14:0_, C_16:0_ N alcohol, C_16:1_ ω*11c* and C_18:1_ ω*9c*. The quinone system contains the major component menaquinone MK-6 and minor component MK-7.

Isolated from various human clinical materials. The type strain is CCM 7927^T^ (= DSM 103682^T^). Sequence accession no. of 16S rRNA gene for the type strain is MH044686. Most characteristics of the type strain CCM 7927^T^ are in agreement with the subspecies description. The strain-dependent test results are as follows: positive β-glucuronidase and negative hydrolysis of DNA and α-glucosidase.

### Description of *macrococcus caseolyticus* subsp. *caseolyticus* subsp. nov.

The subspecies name is created automatically with the same authors as those of *Macrococcus caseolyticus* subsp. *hominis* subsp. nov., in accordance with Rule 40d (formerly Rule 46) of the Prokaryotic Code (De Vos and Trüper, 2000; Parker et al., 2015). The description of *M. caseolyticus* subsp. *caseolyticus* subsp. nov. is based on properties reported previously (Schleifer et al., 1982; Kloos et al., 1998; Mannerová et al., 2003; Schleifer, 2015; Gobeli Brawand et al., 2017) and on phenotypic data determined from the five reference strains included in this study. All strains are gelatin hydrolysis-positive and produce esterase (C4), esterase-lipase (C8) and naphthol-AS-BI-phosphohydrolase. Acid from gluconate, leucine arylamidase and α-glucosidase is variable. Grows in presence of up to 12% NaCl, hydrolysis of Tween 80 and esculin, lipase (C14), valine arylamidase, cysteine arylamidase, trypsin, α-galactosidase, N-acetyl-β-glucosaminidase, α-mannosidase and α-fucosidase negative. Acid production from erythritol, D-arabinose, L-xylose, D-adonitol, β-methyl-D-xyloside, L-sorbose, L-rhamnose, dulcitol, inositol, α-methyl-D-mannoside, α-methyl-D-glucoside, N-acetyl glucosamine, amygdaline, arbutine, D-melibiose, inulin, starch, glycogen, xylitol, gentiobiose, D-lyxose, D-tagatose, D-fucose, L-fucose, D-arabitol, L-arabitol, 2 keto-gluconate and 5 keto-gluconate negative.

The type strain CCM 3540^T^ (= ATCC 13548^T^ = CCUG 15606^T^ = CIP 100755^T^ = DSM 20597^T^) was isolated from cow's milk (Schleifer et al., 1982).

### Description of *macrococcus goetzii* sp. nov.

*Macrococcus goetzii* sp. nov. (goe'tzi.i N.L. masc. gen. n. *goetzii*, of Goetz, named in honor of Prof. Dr. Friedrich Götz, a German microbiologist, for his contribution to the microbiology, physiology, and molecular biology of staphylococci and macrococci).

Cells are Gram-stain positive irregular spherical cocci, occurring predominantly singly and in clusters, non-spore forming and non-motile. Colonies on TSA agar are circular, with whole margins, slightly convex, smooth, shiny, 1–2 mm in diameter, aerobic and non-pigmented. No haemolytic activity. Grows in the presence of up to 7.5% NaCl (weak), at 10°C and 45°C but not at 5°C or 48°C. No growth in the presence of 10% or more NaCl. Weak growth in thioglycolate medium. Catalase, oxidase, nitrate reduction, Voges-Proskauer test (acetoin) and hydrolysis of gelatin positive. Coagulase, clumping factor, urease, arginine dihydrolase, ornithine decarboxylase and pyrrolidonyl arylamidase negative. Susceptible to furazolidon (100 μg) and resistant to novobiocin (5 μg) and bacitracin (10 IU). Hydrolysis of esculin, DNA and Tween 80 negative. Acid phosphatase, alkaline phosphatase, esterase (C4), esterase lipase (C8) (weak), chymotrypsin (weak), naphthol-AS-Bi-phosphohydrolase, β-galactosidase (weak) and α-glucosidase (weak) positive. Lipase (C14), leucine arylamidase, valine arylamidase, cystine arylamidase, trypsin, α-galactosidase, β-glucuronidase, β-glucosidase, N-acetyl-β-glucosaminidase, α-mannosidase and α-fucosidase negative. Acid is produced from glycerol, L-arabinose, galactose (weak), D-glucose, D-fructose, arbutin, salicin, maltose, sucrose, trehalose, melezitose, β-gentiobiose and D-turanose. Acid is not produced from erythritol, D-arabinose, ribose, D-xylose, L-xylose, adonitol, β-methyl-D-xyloside, mannose, sorbose, rhamnose, dulcitol, inositol, mannitol, sorbitol, α-methyl-D-mannoside, α-methyl-D-glucoside, N-acetyl glucosamine, amygdaline, cellobiose, lactose, melibiose, inulin, D-raffinose, starch, glycogen, xylitol, D-lyxose, D-tagatose, D-fucose, L-fucose, D-arabitol, L-arabitol, gluconate, 2 keto-gluconate and 5 keto-gluconate. Susceptible to cefoxitin, cephalothin, ciprofloxacin, erythromycin, gentamicin, chloramphenicol, imipenem, kanamycin, neomycin, sulphamethox/trimethoprim (cotrimoxazol), tetracycline and vancomycin. Resistant to ampicillin, clindamycin, oxacillin and penicillin G. The DNA G+C content of the type strain is 33.95 mol% (calculated from WGS). The peptidoglycan type is A3α L-Lys-Gly_1−2_. The major fatty acids are C_14:0_, C_15:0_ anteiso, C_16:1_ ω*11c*, C_16:0_, and C_18:1_ ω*9c*. The quinone system contains the major component menaquinone MK-6 and minor components MK-5 and MK-7.

The type strain is CCM 4927^T^ (= DSM 103683^T^). Isolated from human clinical material (swab, nail, mycosis). Sequence accession no. of 16S rRNA gene for the type strain is MH044690.

### Description of *macrococcus epidermidis* sp. nov.

*Macrococcus epidermidis* sp. nov. (e.pi.der'mi.dis. N.L. n. *epidermis*, outer skin (from Gr. pref. *epi* and Gr. n. *derma*, skin; Gr. n. *epidermis, -idos*, the outer skin); N.L. gen. n. *epidermidis*, of the outer skin).

Cells are Gram-stain positive spherical cocci, occurring predominantly in pairs and clusters, non-spore forming and non-motile. Colonies on TSA agar are circular, with whole margins, convex with condensed centers, lustreless, 1–2 mm in diameter, aerobic and non-pigmented. No haemolytic activity. Grows in the presence of up to 7.5% NaCl (weak), at 10 and 42°C but not at 5 or 45°C. No growth in the presence of 10% or more NaCl. Weak growth in thioglycolate medium. Catalase, oxidase, nitrate reduction, Voges-Proskauer test (acetoin) and hydrolysis of gelatin positive. Coagulase, clumping factor, urease, arginine dihydrolase, ornithine decarboxylase and pyrrolidonyl arylamidase negative. Susceptible to furazolidon (100 μg) and resistant to novobiocin (5 μg) and bacitracin (10 IU). Hydrolysis of esculin, DNA and Tween 80 negative. Acid phosphatase, alkaline phosphatase, esterase (C4), esterase lipase (C8) (weak), α-glucosidase (weak) and naphthol-AS-Bi-phosphohydrolase positive. Lipase (C14), leucine arylamidase, valine arylamidase, cystine arylamidase, trypsin, α-galactosidase, β-galactosidase, β-glucuronidase, β-glucosidase, N-acetyl-β-glucosaminidase, α-mannosidase and α-fucosidase negative. Acid is produced from glycerol (weak), galactose, D-glucose, D-fructose, N-acetyl glucosamine, arbutin, salicin, maltose, lactose, sucrose, trehalose, melezitose, β-gentiobiose and D-turanose. Acid is not produced from erythritol, D-arabinose, L-arabinose, ribose, D-xylose, L-xylose, adonitol, β-methyl-D-xyloside, mannose, sorbose, rhamnose, dulcitol, inositol, mannitol, sorbitol, α-methyl-D-mannoside, α-methyl-D-glucoside, amygdaline, cellobiose, melibiose, inulin, D-raffinose, starch, glycogen, xylitol, D-lyxose, D-tagatose, D-fucose, L-fucose, D-arabitol, L-arabitol, gluconate, 2 keto-gluconate and 5 keto-gluconate. Susceptible to ampicillin, cefoxitin, cephalothin, ciprofloxacin, erythromycin, gentamicin, chloramphenicol, imipenem, kanamycin, neomycin, oxacillin, penicillin G, sulphamethox/trimethoprim (cotrimoxazol), tetracycline and vancomycin. Resistant to clindamycin. The DNA G+C content of the type strain is 33.97 mol% (calculated from WGS). The peptidoglycan type is A3α Lys-Gly_3_-Ser. The major fatty acids are C_15:0_ anteiso, C_18:1_ ω*9c* and C_18:0_. Quinone system contains the major component menaquinone MK-6 and minor component MK-7.

The type strain is CCM 7099^T^ (= DSM 103681^T^). Isolated from human clinical material (swab, mycosis). Sequence accession no. of 16S rRNA gene for the type strain is MH044691.

### Description of *macrococcus bohemicus* sp. nov.

*Macrococcus bohemicus* sp. nov. (bo.he'mi.cus. N.L. masc. adj. *bohemicus*, pertaining to Bohemia, a region of the Czech Republic where the type strain was isolated).

Cells are Gram-stain positive spherical cocci, occurring predominantly in pairs and tetrads, non-spore forming and non-motile. Colonies on TSA agar are circular, with whole margins, flat with convex center, lustreless, 1-2 mm in diameter, aerobic and nonpigmented. No haemolytic activity. Grows in the presence of 7.5% NaCl, at 10°C and 42°C but not at 5 or 45°C. Growth in thioglycolate medium. No growth in the presence of 10% or more NaCl. Catalase, oxidase, nitrate reduction, Voges-Proskauer test (acetoin) and hydrolysis of gelatin positive. Coagulase, clumping factor, urease, arginine dihydrolase, ornithine decarboxylase and pyrrolidonyl arylamidase negative. Susceptible to furazolidon (100 μg) and resistant to novobiocin (5 μg) and bacitracin (10 IU). Hydrolysis of esculin, DNA and Tween 80 negative. Acid phosphatase (weak), esterase (C4), esterase lipase (C8), chymotrypsin and naphthol-AS-Bi-phosphohydrolase positive. Lipase (C14), alkaline phosphatase, leucine arylamidase, valine arylamidase, cystine arylamidase, trypsin, α-galactosidase, β-galactosidase, β-glucuronidase, α-glucosidase β-glucosidase, N-acetyl-β-glucosaminidase, α-mannosidase and α-fucosidase negative. Acid is produced from glycerol (weak), galactose, D-glucose, D-fructose, N-acetyl glucosamine, arbutin, salicin, maltose, lactose, sucrose, trehalose, D-raffinose (weak), β-gentiobiose and D-turanose (weak). Acid is not produced from erythritol, D-arabinose, L-arabinose, ribose, D-xylose, L-xylose, adonitol, β-methyl-D-xyloside, mannose, sorbose, rhamnose, dulcitol, inositol, mannitol, sorbitol, α-methyl-D-mannoside, α-methyl-D-glucoside, amygdaline, cellobiose, melibiose, inulin, melezitose, starch, glycogen, xylitol, D-lyxose, D-tagatose, D-fucose, L-fucose, D-arabitol, L-arabitol, gluconate, 2 keto-gluconate and 5 keto-gluconate. Susceptible to ampicillin, cefoxitin, cephalothin, ciprofloxacin, erythromycin, gentamicin, chloramphenicol, imipenem, kanamycin, neomycin, oxacillin, penicillin G, sulphamethox/trimethoprim (cotrimoxazol), tetracycline and vancomycin. Resistant to clindamycin. The DNA G+C content of the type strain is 34.9 mol% (calculated from WGS). Peptidoglycan type is A3α L-Lys-Gly_1−2_. Major fatty acids are C_15:0_ anteiso, C_16:0_ and C_18:1_ ω*9c*. Quinone system contains major component menaquinone MK-6 and minor components MK-5 and MK-7.

The type strain is CCM 7100^T^ (= DSM 103680^T^). Isolated from human clinical material (wound, knee). Sequence accession no. of 16S rRNA gene for the type strain is MH044692.

### Emended description of genus *macrococcus* kloos, ballard, george, webster, hubner, ludwig, schleifer, fiedler and schubert 1998

The phenotypic characteristics are identical to those of the original genus description and additional references (Mannerová et al., 2003; Gobeli Brawand et al., 2017) and the above-mentioned data in subspecies descriptions, except for the following: DNA G+C content is in the range 34–39 mol%.

## Author contributions

RP, IS, and IM designed the study. ZW, IS, PŠ, PS, CS, SK, OŠ, LK, VV, TF, and PP performed the experiments; IM, ZW, AI, VK, and OŠ analyzed the data; ZZ, VR and JD contributed the theory and experimental design; IM, ZW, AI, IS, and RP wrote the paper.

### Conflict of interest statement

The authors declare that the research was conducted in the absence of any commercial or financial relationships that could be construed as a potential conflict of interest.
