# Supplementary material for: Description and Comparative Genomics of Macrococcus caseolyticus subsp. hominis subsp. nov., Macrococcus goetzii sp. nov., Macrococcus epidermidis sp. nov., and Macrococcus bohemicus sp. nov., Novel Macrococci From Human Clinical Material With Virulence Potential and Suspected Uptake of Foreign DNA by Natural Transformation
Source: Front Microbiol. 2018 Jun 13;9:1178. doi: 10.3389/fmicb.2018.01178 (PMC6008420; doi:10.3389/fmicb.2018.01178)
Supplement: Supplementary file 1 [file Table_1.DOCX]

**TABLE S1.** Cellular fatty acid composition (as a percentage of the total) of *M. caseolyticus* subsp. *hominis* (CCM 7927^T^, CCM 7928, P862, P865), *M. goetzii* CCM 4927^T^, *M. epidermidis* CCM 7099^T^, and *M. bohemicus* CCM 7100^T^. Values of less than 1 % are not shown.

| **Fatty acid** | **CCM 4927^T^** | **CCM 7099^T^** | **CCM 7100^T^** | **CCM 7927^T^** | **CCM 7928** | **P862** | **P865** | ***CCM 3540^T^** | ***KM 45013^T^** |
| --- | --- | --- | --- | --- | --- | --- | --- | --- | --- |
| C_13:0_ iso | 2.4 | 1.2 | 1.3 | ND | ND | ND | ND | ND | NA |
| C_13:0_ anteiso | 1.5 | 1.0 | 1.1 | ND | ND | ND | ND | ND | NA |
| C_14:0_ iso | 3.4 | 5.3 | 7.7 | 1.0 | 1.0 | 1.0 | 1.4 | 7.9 | 1.2 |
| C_14:0_ | **11.8** | 3.6 | 8.1 | **27.4** | **27.1** | **27.2** | **26.5** | 16.5 | 29.5 |
| C_15:0_ iso | 5.5 | 4.8 | 4.1 | ND | ND | ND | ND | ND | NA |
| C_15:0_ anteiso | **12.3** | **17.5** | **15.2** | ND | ND | ND | ND | ND | NA |
| C_16:1_ *ω7c* alcohol | TR | TR | 1.1 | ND | ND | ND | ND | TR | NA |
| C_16:0_ N alcohol | TR | 1.6 | 2.6 | **14.2** | **14.2** | **13.8** | **12.3** | ND | 20.7 |
| C_16:1_ *ω11c* | **11.8** | 6.0 | 8.0 | **11.5** | **12.1** | **11.3** | **11.1** | 29.5 | 18.4 |
| C_16:0_ | **9.9** | 8.8 | **14.9** | **9.6** | **8.9** | **9.8** | **10.4** | 6.6 | 5.0 |
| C_17:0_ anteiso | ND | 1.1 | 1.0 | ND | ND | ND | ND | ND | NA |
| C_17:1_ *ω10c* | ND | ND | ND | ND | ND | ND | ND | ND | ND |
| C_18:3_ *ω6c* (6,9,12) | TR | 2.8 | 2.3 | **6.7** | **6.5** | **6.6** | **6.8** | ND | 22.3 |
| C_18:1_ *ω9c* | **31.5** | **27.6** | **20.2** | **22.2** | **23.6** | **22.4** | **23.2** | 32.6 | NA |
| C_18:0_ | 6.1 | **12.7** | 8.5 | 4.9 | ND | 5.2 | 4.9 | 1.4 | 1.5 |
| C_20:0_ | TR | 1.0 | TR | 1.3 | 1.2 | 1.2 | 1.4 | ND | 0.7 |
| C_20:1_ *ω9c* | ND | ND | ND | TR | TR | TR | ND | 2.0 | NA |

*Data of *M. canis* KM 45013^T^ (Brawand et al., 2016), *M. caseolyticus* CCM 3540^T^ (Mannerová et al., 2003).

Legend: ND, not detected; TR, trace amounts <1%; NA, not analyzed; bold font, major fatty acids
