# Supplementary material for: Description and Comparative Genomics of Macrococcus caseolyticus subsp. hominis subsp. nov., Macrococcus goetzii sp. nov., Macrococcus epidermidis sp. nov., and Macrococcus bohemicus sp. nov., Novel Macrococci From Human Clinical Material With Virulence Potential and Suspected Uptake of Foreign DNA by Natural Transformation
Source: Front Microbiol. 2018 Jun 13;9:1178. doi: 10.3389/fmicb.2018.01178 (PMC6008420; doi:10.3389/fmicb.2018.01178)
Supplement: Supplementary file 3 [file Image_1.PDF]

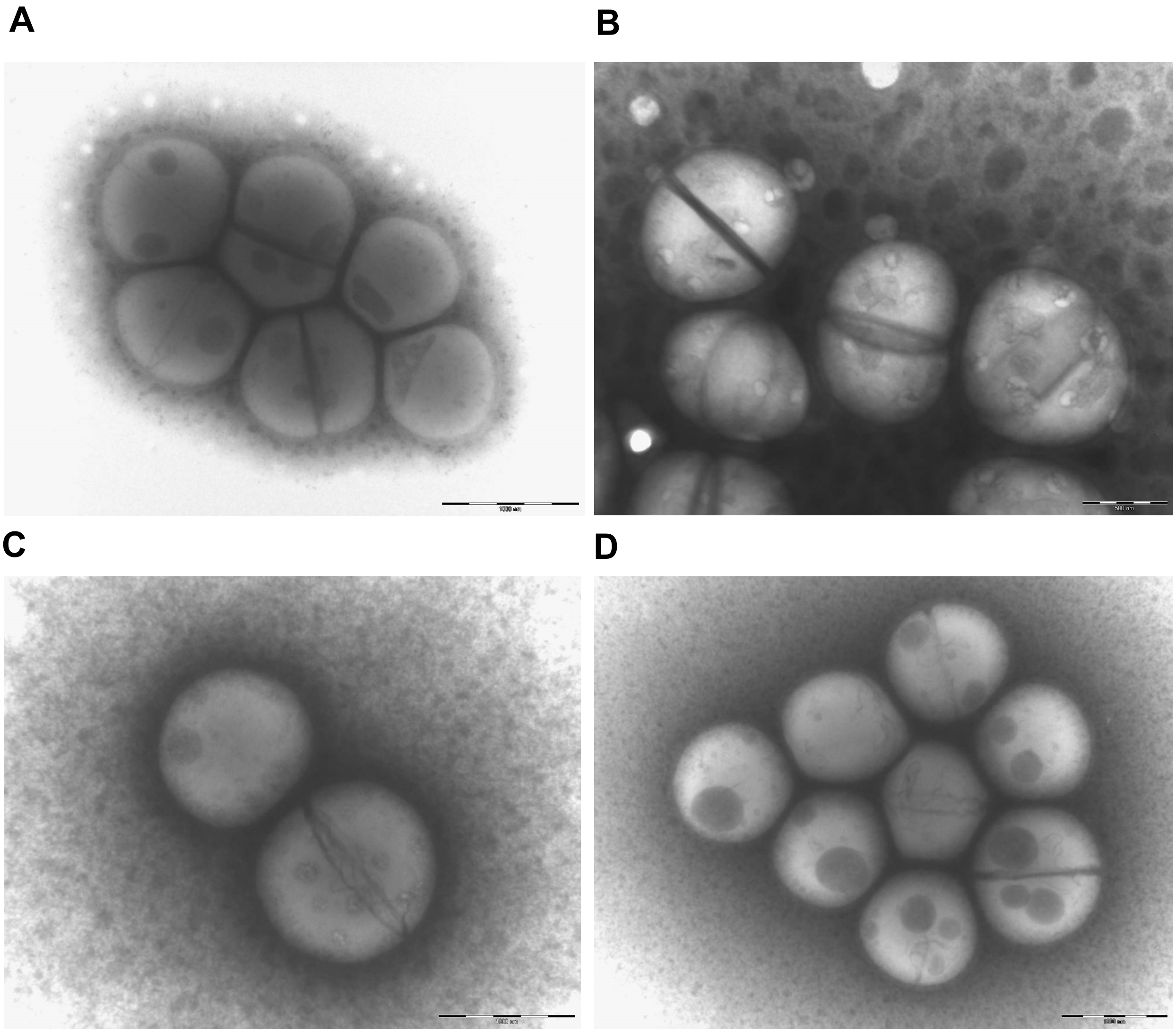

FIGURE S1. Transmission electron microscopy of cells of *Macrococcus* spp. performed with Morgagni 268D Philips (FEI Company, USA) electron microscope. Negative staining with 2% ammonium molybdate. **(A)** *M. caseolyticus* subsp. *hominis* CCM 7927 ^{T}, **(B)** *M. goetzii* CCM 4927^{T}, **(C)** *M. epidermidis* CCM 7099^{T}, **(D)** *M. bohemicus* CCM 7100^{T}. Bar represents 1000 nm (A, C, and D) or 500 nm (B).
