# Supplementary material for: Description and Comparative Genomics of Macrococcus caseolyticus subsp. hominis subsp. nov., Macrococcus goetzii sp. nov., Macrococcus epidermidis sp. nov., and Macrococcus bohemicus sp. nov., Novel Macrococci From Human Clinical Material With Virulence Potential and Suspected Uptake of Foreign DNA by Natural Transformation
Source: Front Microbiol. 2018 Jun 13;9:1178. doi: 10.3389/fmicb.2018.01178 (PMC6008420; doi:10.3389/fmicb.2018.01178)
Supplement: Supplementary file 4 [file Image_2.PDF]

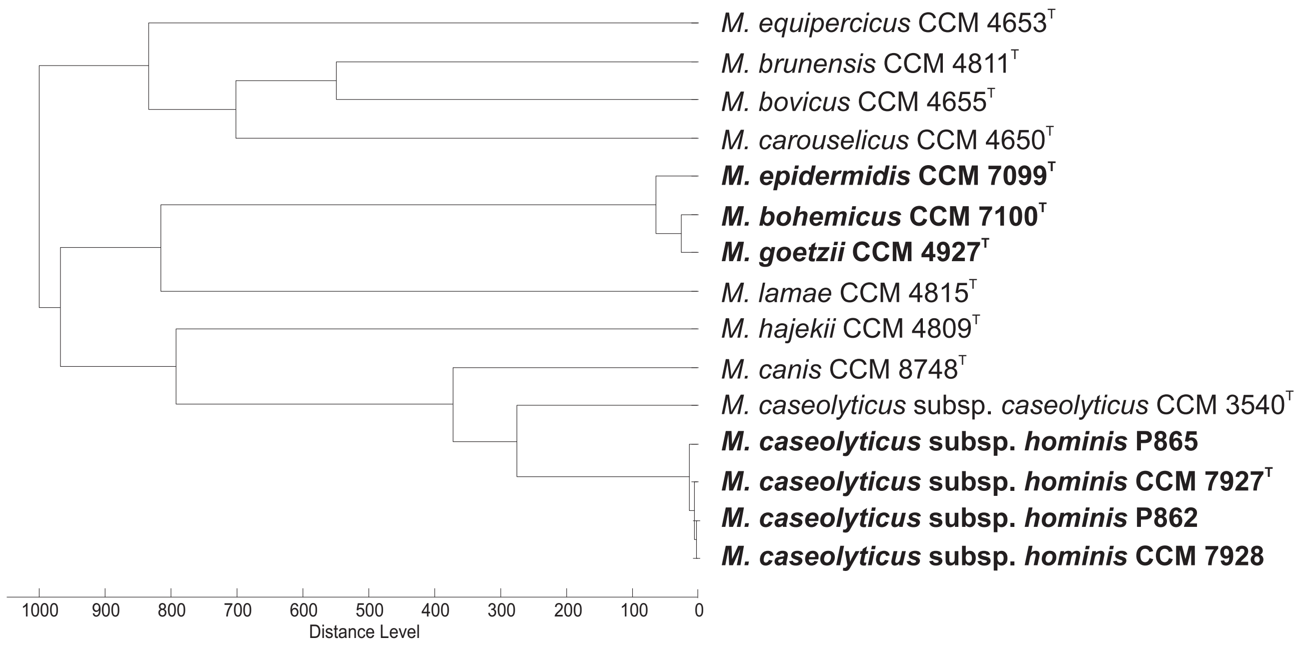

FIGURE S2. Dendogram based on MALDI-TOF mass specra of the 15 *Macrococcus* spp. strains obtained using ferulic acid as a MALDI matrix. The MALDI-TOF MS profiles were acquired using an Ultraflextreme instrument (Bruker Daltonik) and the dendrogram was generated using the correlation distance measure with the average linkage algorithm (UPGMA).
