# Supplementary material for: Description and Comparative Genomics of Macrococcus caseolyticus subsp. hominis subsp. nov., Macrococcus goetzii sp. nov., Macrococcus epidermidis sp. nov., and Macrococcus bohemicus sp. nov., Novel Macrococci From Human Clinical Material With Virulence Potential and Suspected Uptake of Foreign DNA by Natural Transformation
Source: Front Microbiol. 2018 Jun 13;9:1178. doi: 10.3389/fmicb.2018.01178 (PMC6008420; doi:10.3389/fmicb.2018.01178)
Supplement: Supplementary file 5 [file Image_3.PDF]

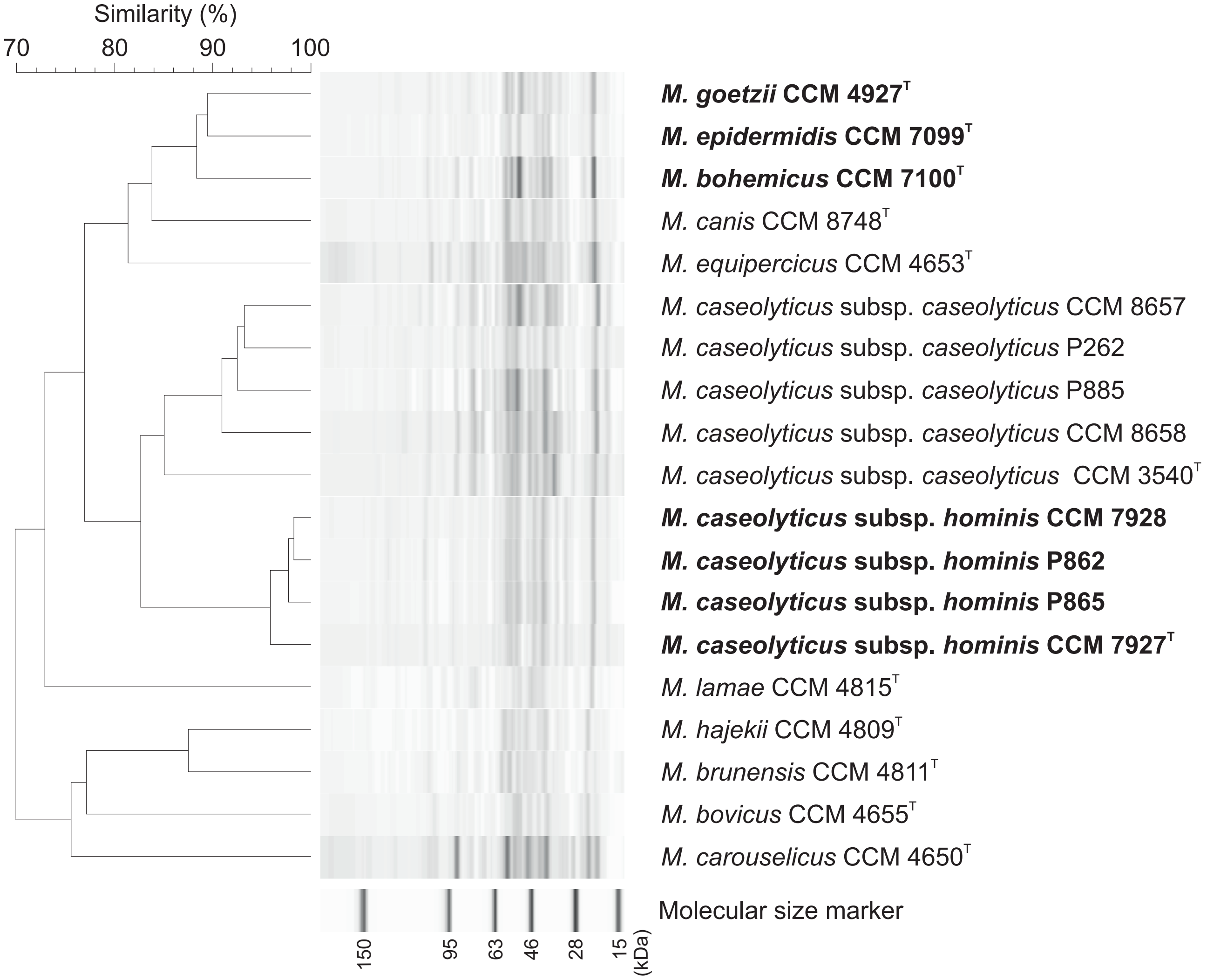

FIGURE S3. Dendrogram based on cluster analysis of whole-cell protein profiles obtained using Agilent 2100 Bioanalyzer system with Protein 230 kit (Agilent Technologies) from investigated strains and representative strains of phylogenetically related *Macrococcus* species. The dendrogram was calculated with Pearson's correlation coefficients by the UPGMA clustering method (*r*, expressed as percentage similarity values).
