# Supplementary material for: Description and Comparative Genomics of Macrococcus caseolyticus subsp. hominis subsp. nov., Macrococcus goetzii sp. nov., Macrococcus epidermidis sp. nov., and Macrococcus bohemicus sp. nov., Novel Macrococci From Human Clinical Material With Virulence Potential and Suspected Uptake of Foreign DNA by Natural Transformation
Source: Front Microbiol. 2018 Jun 13;9:1178. doi: 10.3389/fmicb.2018.01178 (PMC6008420; doi:10.3389/fmicb.2018.01178)
Supplement: Supplementary file 7 [file Image_5.PDF]

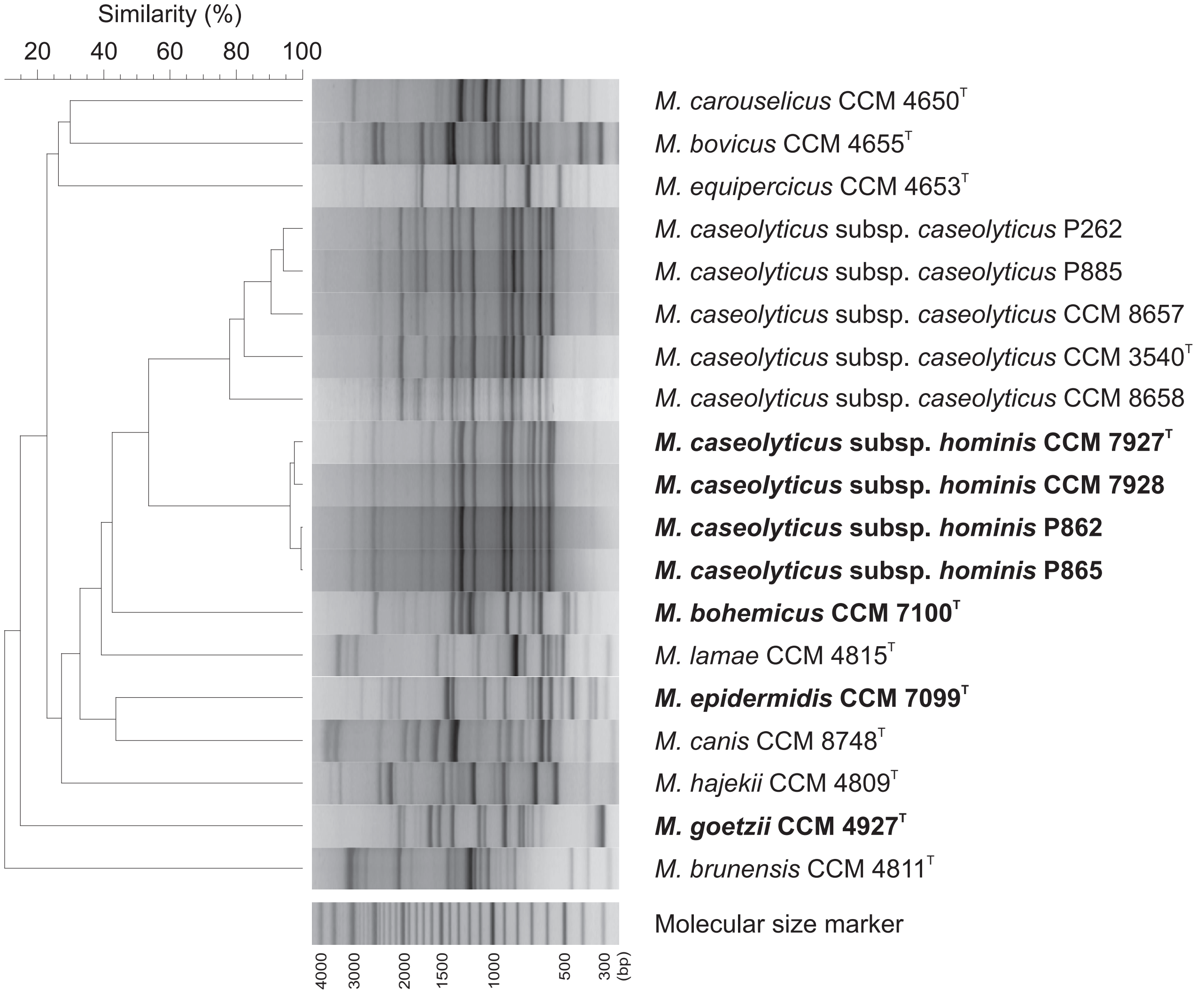

FIGURE S5. Dendrogram based on cluster analysis of rep-PCR fingerprints obtained with $(GTG)_5$ primer from investigated strains and representative strains of phylogenetically related *Macrococcus* species. The dendrogram was calculated with Pearson's correlation coefficients by the UPGMA clustering method (*r*, expressed as percentage similarity values).
