# Supplementary material for: Description and Comparative Genomics of Macrococcus caseolyticus subsp. hominis subsp. nov., Macrococcus goetzii sp. nov., Macrococcus epidermidis sp. nov., and Macrococcus bohemicus sp. nov., Novel Macrococci From Human Clinical Material With Virulence Potential and Suspected Uptake of Foreign DNA by Natural Transformation
Source: Front Microbiol. 2018 Jun 13;9:1178. doi: 10.3389/fmicb.2018.01178 (PMC6008420; doi:10.3389/fmicb.2018.01178)
Supplement: Supplementary file 8 [file Image_6.PDF]

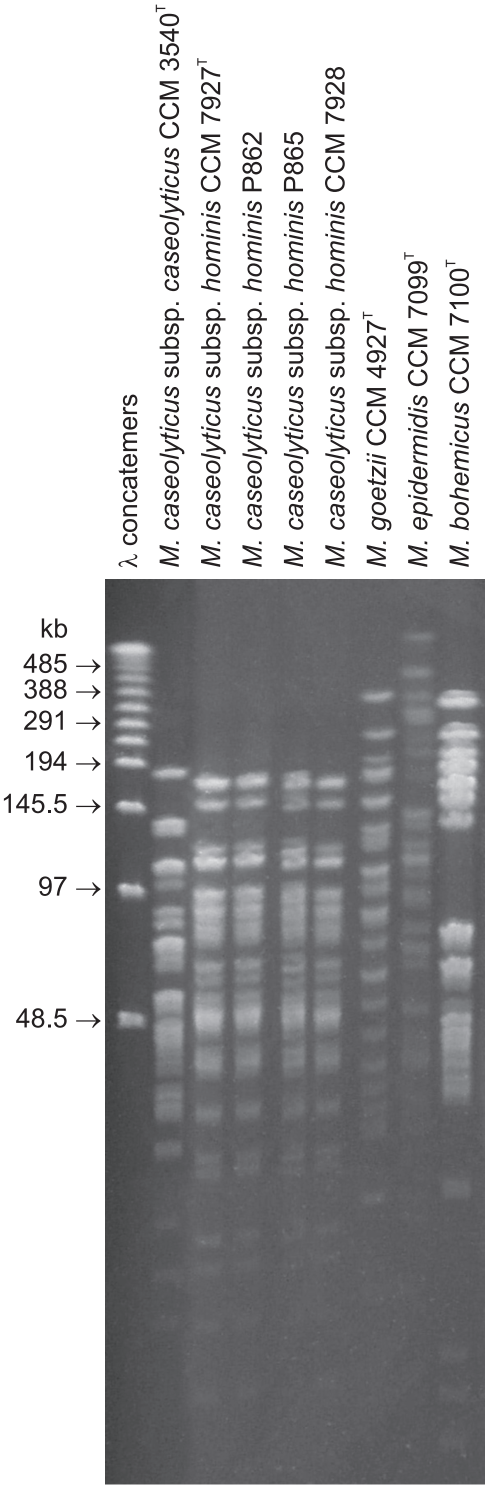

FIGURE S6. Pulsed-field gel electrophoresis showing macrorestriction patterns of *Sma*I-digested chromosomal DNAs of analyzed *Macrococcus* spp. Concatemers of DNA from phage λ were used as a size marker (lane 1).
