# Supplementary material for: Description and Comparative Genomics of Macrococcus caseolyticus subsp. hominis subsp. nov., Macrococcus goetzii sp. nov., Macrococcus epidermidis sp. nov., and Macrococcus bohemicus sp. nov., Novel Macrococci From Human Clinical Material With Virulence Potential and Suspected Uptake of Foreign DNA by Natural Transformation
Source: Front Microbiol. 2018 Jun 13;9:1178. doi: 10.3389/fmicb.2018.01178 (PMC6008420; doi:10.3389/fmicb.2018.01178)
Supplement: Supplementary file 9 [file Image_7.PDF]

A

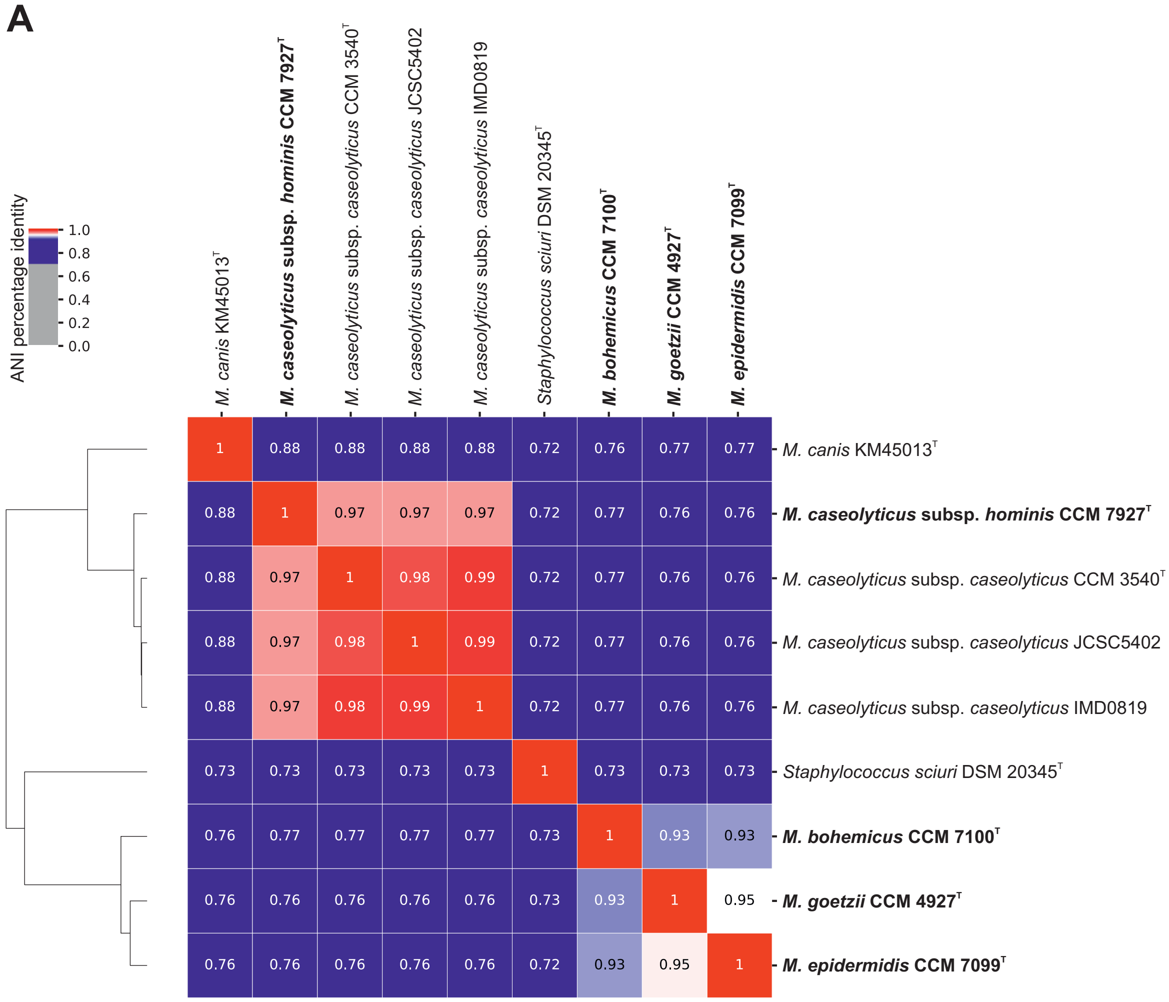

B

Reference	*M. caseolyticus* subsp. *hominis* CCM 7927^T		*M. goetzii* CCM 4927^T		*M. epidermidis* CCM 7099^T		*M. bohemicus* CCM 7100^T	
	DDH (%)	GGDC (%)	DDH (%)	GGDC (%)	DDH (%)	GGDC (%)	DDH (%)	GGDC (%)
CCM 3540^{T*}	87	72.80 +/- 2.92	14	20.50 +/- 2.32	29	20.40 +/- 2.32	19	22.00 +/- 2.35
CCM 7927^T			19	21.00 +/- 2.33	15	20.40 +/- 2.32	27	22.40 +/- 2.36
CCM 4927^T					49	62.40 +/- 2.85	45	49.70 +/- 2.63
CCM 7099^T							56	52.00 +/- 2.67

* *M. caseolyticus* subsp. *caseolyticus*

FIGURE S7. Results of genome sequence-based species delimitation compared to the DNA-DNA hybridization (DDH) results. **(A)** Heat map of average nucleotide identity (ANI) values amongst phylogenetically related *Macrococcus* species. GenBank accession numbers of used reference genomes are as follows: *M. caseolyticus* JCSC5402 (AP009484), *M. caseolyticus* IMD0819 (CP021058), *M. canis* KM45013^T (CP021059), and *S. sciuri* DSM 20345^T (NZ_LEOS00000000). Red indicates identity above 95 % threshold. **(B)** The DNA-DNA hybridization (DDH) and digitally derived genome-to-genome distances emulating DNA-DNA hybridization calculated using GGDC tool available at http://ggdc.dsmz.de/. Formula no. 2 recommended for draft genomes was used for interpretation of the results. Red indicates identity above 70 % threshold.
